# Biomolecular solid-state NMR spectroscopy at 1200 MHz: the gain in resolution

**DOI:** 10.1007/s10858-021-00373-x

**Published:** 2021-06-25

**Authors:** Morgane Callon, Alexander A. Malär, Sara Pfister, Václav Římal, Marco E. Weber, Thomas Wiegand, Johannes Zehnder, Matías Chávez, Riccardo Cadalbert, Rajdeep Deb, Alexander Däpp, Marie-Laure Fogeron, Andreas Hunkeler, Lauriane Lecoq, Anahit Torosyan, Dawid Zyla, Rudolf Glockshuber, Stefanie Jonas, Michael Nassal, Matthias Ernst, Anja Böckmann, Beat H. Meier

**Affiliations:** 1 Physical Chemistry, ETH Zurich, 8093 Zurich, Switzerland; 2 Molecular Microbiology and Structural Biochemistry, UMR 5086 CNRS, Université de Lyon, 69367 Lyon, France; 3 Institute of Molecular Biology and Biophysics, ETH Zurich, 8093 Zurich, Switzerland; 4 Department of Medicine II / Molecular Biology, University of Freiburg, Freiburg im Breisgau, Germany

**Keywords:** Solid-state NMR, Magic-angle spinning, Biomolecular NMR, High field, Helicases, Viruses

## Abstract

Progress in NMR in general and in biomolecular applications in particular is driven by increasing magnetic-field strengths leading to improved resolution and sensitivity of the NMR spectra. Recently, persistent superconducting magnets at a magnetic field strength (magnetic induction) of 28.2 T corresponding to 1200 MHz proton resonance frequency became commercially available. We present here a collection of high-field NMR spectra of a variety of proteins, including molecular machines, membrane proteins, viral capsids, fibrils and large molecular assemblies. We show this large panel in order to provide an overview over a range of representative systems under study, rather than a single best performing model system. We discuss both carbon-13 and proton-detected experiments, and show that in ^13^C spectra substantially higher numbers of peaks can be resolved compared to 850 MHz while for ^1^H spectra the most impressive increase in resolution is observed for aliphatic side-chain resonances.

**Supplementary Information:**

The online version contains supplementary material available at 10.1007/s10858-021-00373-x

## Introduction

New technologies have often stood at the beginning of new spectroscopic techniques and NMR is a particularly good example: Microcomputers have enabled Fourier spectroscopy (Ernst and Anderson 1965) and multidimensional NMR (Aue et al. 1976), high and stable magnetic fields generated by persistent superconducting magnets have been instrumental for the first protein structure determinations (Williamson et al. 1985; Wüthrich 2003) and the structural and dynamic investigation of increasingly larger proteins (Pervushin et al. 1997; Fiaux et al. 2002; Rosenzweig and Kay 2014). Reliable magic-angle sample spinning probes together with high magnetic fields have enabled biomolecular solid-state NMR spectroscopy (McDermott et al. 2000). The first solid-state NMR protein-structure determination used a magnetic-field strength of 17.6 T (proton resonance frequency 750 MHz) (Castellani et al. 2002), and the first prion fibril structure was determined at 850 MHz (Wasmer et al. 2008). A next achievement with important impact was the development of fast magic-angle spinning (MAS) probes, in excess of 100 kHz rotation frequency, enabling proton detection and a reduction of the required sample amount by roughly two orders of magnitude (Barbet-Massin et al. 2014; Agarwal et al. 2014; Andreas et al. 2015; Lecoq et al. 2019; Penzel et al. 2019; Schledorn et al. 2020).

Since 1000 MHz proton Larmor frequency is the present limit of what could be achieved with low-temperature superconducting (LTS) wire (such as Nb_3_Sn and NbTi), persistent magnetic fields exceeding 1000 MHz required solenoid coils made out of high-temperature superconducting (HTS) wire (e.g. REBCO) (Maeda and Yanagisawa 2019). Thus, after the highest LTS magnet (1 GHz), it has taken more than 5 years to develop this new technology and achieve higher fields. Today, persistent hybrid superconducting magnets combining both, LTS and HTS, have been developed by Bruker Switzerland AG generating magnetic-field strengths up to 28.2 T corresponding to 1200 MHz proton Larmor frequency.

What improvement in resolution and sensitivity do we expect by an increase in magnetic field from 850 to 1200 MHz? Assuming that the NMR linewidths are dominated by small scalar couplings, as in our examples of carbon-detected spectroscopy, they should be field-independent when expressed in frequency units (Hz). If it is determined by dipolar interactions coming from second-order average Hamiltonian terms, often encountered in proton detection, they are at least independent of the field strength, but can even decrease due to the reduction of these terms by the larger chemical-shift differences (more towards a weak coupling situation) (Malär et al. 2019a). In both cases, resolution in NMR spectra benefits when going from 850 to 1200 MHz through an increase in chemical-shift dispersion (in Hz) by a factor of nearly 1.5 (the ratio of the two magnetic fields). On the ppm scale, the linewidth decreases linearly (or somewhat stronger) with increasing *B*_0_ by the same factor of around 1.5 (see Figure S1 for an illustration). With respect to sensitivity, the theoretical gain in signal-to-noise ratio (SNR) is given by ${\left(\frac{{B}_{\text{0,1200}}}{{B}_{\text{0,850}}}\right)}^{3/2}$ (Abragam 1961), which corresponds to a factor of 1.7 in the integral of the peaks. These considerations apply both to ^13^C- and ^1^H-detected experiments.

The above values are valid for “perfect” samples, which do neither show conformational disorder (resulting in heterogenous line broadening), nor dynamics (resulting in homogenous line broadening). Heterogeneous line broadening (in Hz) increases linearly with the magnetic field. Expressed in ppm, this contribution to the total linewidth is independent of *B*_0_ and so is the spectral resolution. In real samples, both disorder and dynamics can represent important contributions to the linewidths; this is why it is important to illustrate the gain achieved for a broad selection of samples. Besides these sample-dependent effects, several instrumental imperfections can limit the quality of the spectra, including magnetic-field inhomogeneity in space (shims) and in time (field drifts), limited rf power (offset effects), or imperfect or unstable magic-angle adjustment and radio-frequency (rf) field inhomogeneity. There are a number of intrinsic challenges when going to higher fields: the larger chemical-shift dispersion makes the application of higher power pulses necessary to cover the entire spectrum; at the same time, obtaining high rf fields becomes more demanding at higher frequency, in particular for lossy samples with a high salt content.

We herein present first results obtained on a 1200 MHz spectrometer for a set of biomolecular samples that we have already investigated at 850 MHz, and compare sensitivity and resolution in ^1^H- and ^13^C-detected NMR spectra. Proton-detected spectra at 1200 MHz are also under investigation in other labs and a preprint has become available during the submission process as (Nimerovsky et al. 2021). We avoided the temptation to select one “typical” sample, i.e. the very best performing sample that we have, but rather present a selection of samples that we are currently investigating in the laboratory. We used both, the more classical approach of ^13^C-detected spectroscopy, which shows higher absolute intensity when large sample quantities (approx. 30 mg) can be prepared (Lecoq et al. 2019; Mandala and Hong 2019), as well as ^1^H-detected solid-state NMR, which has a mass sensitivity about 50 times higher, and relies on the use of sub milligram protein quantities (Agarwal et al. 2014; Lecoq et al. 2019). Both approaches are today central in biomolecular NMR spectroscopy, and show different hardware requirements, sample amounts, isotope-labelling protocols as well as strengths and limitations with regard to the information contents of the fingerprint spectra obtained (^13^C–^13^C correlation vs. ^15^N–^1^H correlation).

## Results

In the following, we compare spectra of amyloid fibrils of the fungal prion HET-s(218–289) (Wasmer et al. 2008; van Melckebeke et al. 2010); sediments of the bacterial helicase DnaB (Gardiennet et al. 2012; Wiegand et al. 2019); the bacterial RNA helicase and acetyltransferase TmcA (Ikeuchi et al. 2008; Chimnaronk et al. 2009); the Rpo4/7 protein complex of two subunits of archaeal RNA polymerase II (Torosyan et al. 2019); the filaments of PYRIN domain of mouse ASC (Sborgi et al. 2015; Ravotti et al. 2016); the viral capsids of the Hepatitis B virus (Lecoq et al. 2019) and the African cichlid nackednavirus (Lauber et al. 2017); supramolecular protein filaments of type 1 pili (Hahn et al. 2002; Habenstein et al. 2015); and the nonstructural membrane protein 4B (NS4B) of the Hepatitis C virus. In all figures, spectra colored in blue were recorded at 850 MHz, and spectra in red at 1200 MHz.

### ^13^C-detected ^13^C–^13^C correlation spectroscopy

**Fig. 1:**
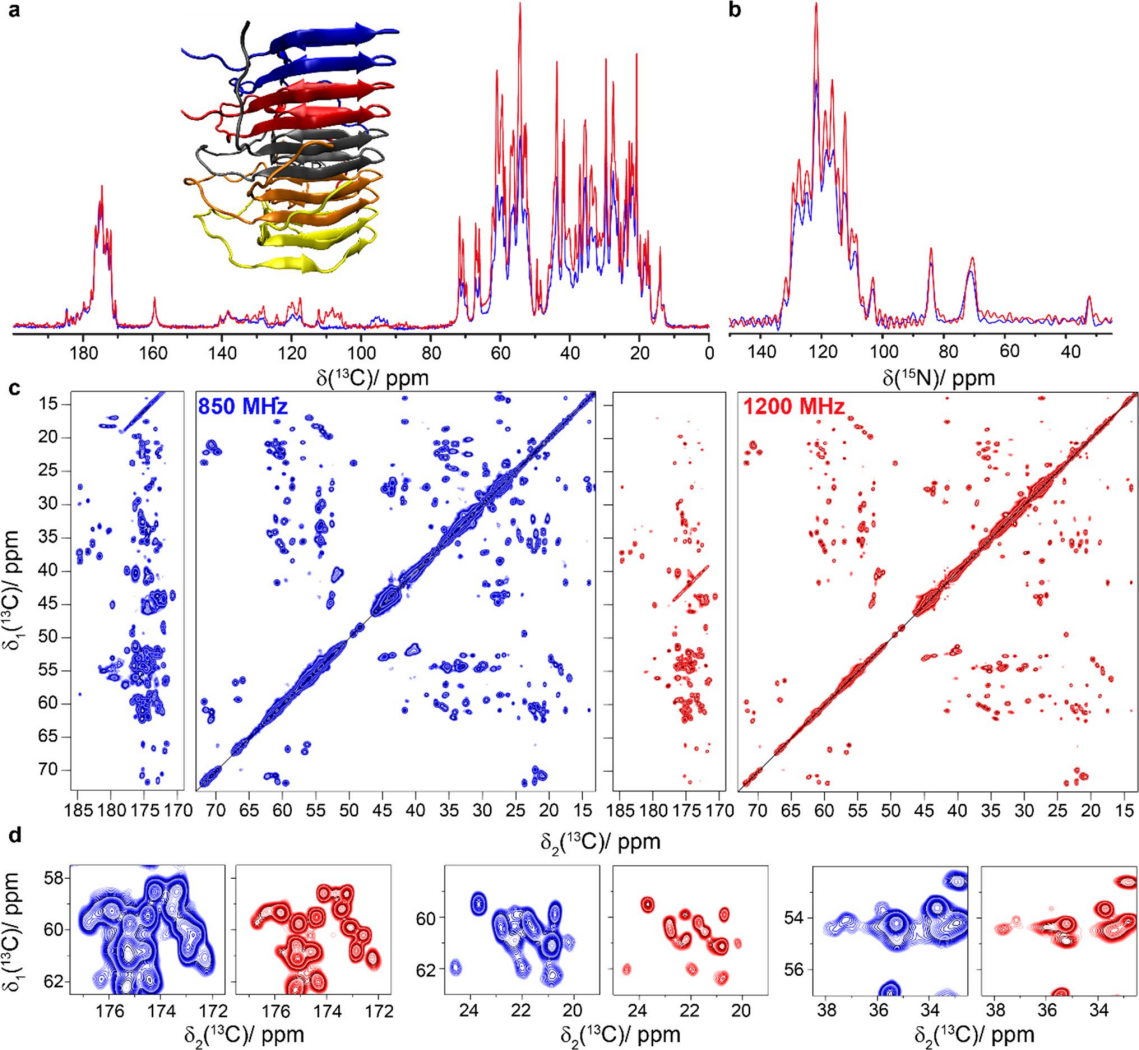
HET-s(218–289) amyloid fibrils. **a** Structure model (PDB ID: 2RNM) (Wasmer et al. 2008) and 1D ^13^C-detected CP-MAS spectrum, **b** 1D ^15^N-detected CP-MAS spectrum, **c** 20 ms DARR spectra and **d** expanded regions from the spectra in **c**. Spectra colored in blue were recorded at 850 MHz and spectra in red were measured at 1200 MHz. CP was matched at 75 and 48 kHz for ^1^H and ^13^C at 1200 MHz and 60 and 43 kHz at 850 MHz. Experimental parameters are listed in Table S1. The two spectra were normalized using isolated well-resolved peaks and the contour levels are the same for the two spectra

Figure 1a and b compare ^13^C- and ^15^N-detected cross-polarization (CP) spectra of HET-s(218–289) amyloid fibrils (Wasmer et al. 2008; van Melckebeke et al. 2010; Smith et al. 2017) measured in a commercial Bruker 3.2 mm triple-resonance probe using the E-free design (Gor’kov et al. 2007) at 850 and 1200 MHz. Out of the 71 residues of HET-s(218–289), 56 are observed in CP spectra (van Melckebeke et al. 2010), the remainder is invisible due to dynamics (Siemer et al. 2006; Smith 2018). A sensitivity gain is observed in the ^13^C spectrum at 1200 MHz in which the aliphatic region is favored. We attribute this observation to the offset dependence of the CP step caused by the limited rf-field strength available at the 1200 MHz spectrometer on the ^13^C channel of the probe (the ~ 48 kHz used are not large compared to the ^13^C spectral width). The aliphatic regions of 2D ^13^C–^13^C Dipolar Assisted Rotational Resonance (DARR) spectra (Takegoshi et al. 2001, 2003) of HET-s(218–289) recorded at 850 and 1200 MHz are given in Fig. 1c, with expanded regions shown in Fig. 1d. It can clearly be seen that the spectra at 1200 MHz show higher resolution. However, one can conclude from the 1D traces (Figure S3) that the DARR transfer is, as expected, somewhat less efficient for constant mixing time at the higher magnetic field. Since the MAS frequency of the 1200 MHz 3.2 mm probe is currently limited to 20 kHz, corresponding to ~ 66 ppm, some rotational-resonance (Colombo et al. 1988; Raleigh et al. 1988) line-broadening effects are present at 1200 MHz between the carbonyl and aliphatic resonances. The linewidth thus can still be improved by spinning faster; a MAS frequency of around 24 kHz would be optimal, and can generally be achieved in 3.2 mm rotors (Böckmann et al. 2015).

**Fig. 2:**
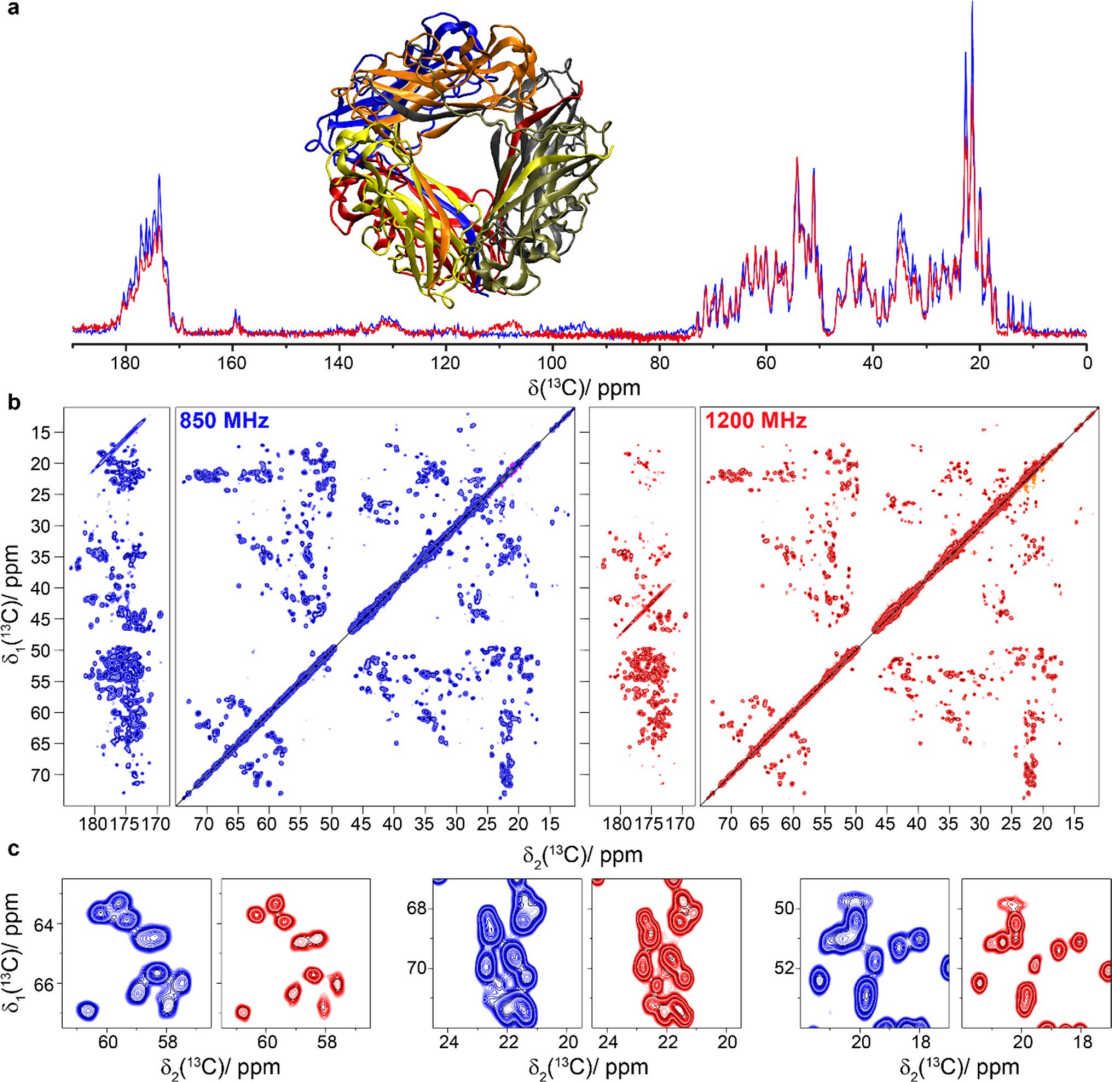
Protein filaments of type 1 pili. **a** Structural model (PDB ID: 2N7H) (Habenstein et al. 2015) and 1D ^13^C-detected CP-MAS spectrum, **b** 20 ms DARR spectra and **c** spectral fingerprints expanded from the spectra in **b**. Spectra colored in blue were recorded at 850 MHz and spectra in red were measured at 1200 MHz. CP was matched at 70 and 44 kHz for ^1^H and ^13^C at 1200 MHz and at 60 and 43 kHz at 850 MHz

As a second system, we show adhesive type 1 pili from *E.coli*, which assemble in vitro to form long supramolecular protein filaments (Hahn et al. 2002; Habenstein et al. 2015). Each monomer consists of 150 amino acids. Using 850 MHz data, the ^13^C resonances have been assigned to 98% of the sequence (Habenstein et al. 2015). A clear improvement in resolution at the higher field is observed in the expanded regions shown in Fig. 2c.

**Fig. 3:**
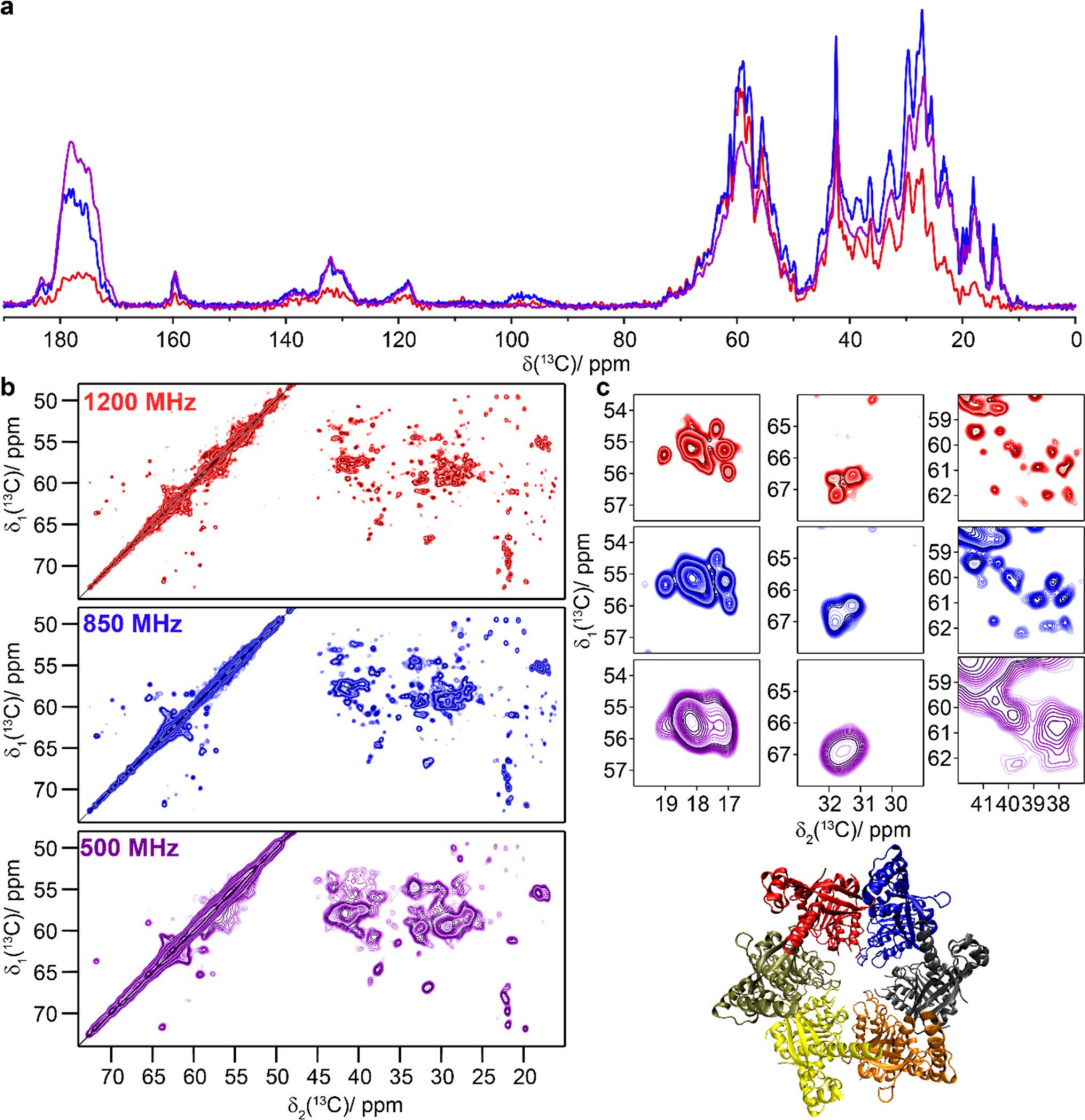
The bacterial DnaB helicase. **a** 1D ^13^C-detected CP-MAS spectra recorded at 500, 850, and 1200 MHz; **b** 20 ms DARR spectra recorded at the same magnetic fields as in **a**. **c** Expanded regions from the spectra in **b**. Spectra colored in purple were measured at 500 MHz, spectra in blue were recorded at 850 MHz and spectra in red were measured at 1200 MHz. CP was matched at 55 and 29 kHz for ^1^H and ^13^C at 1200 MHz and at 60 and 43 kHz at 500 and 850 MHz. The 1D spectra in **a** were scaled to a similar noise level. Structural model with each subunit colored differently (PDB ID: 4ZC0) (Bazin et al. 2015)

While the type 1 pili and HET-s(218–289) were small enough for assignment and structure determination at 850 MHz (Wasmer et al. 2008; van Melckebeke et al. 2010) (Habenstein et al. 2015), the DnaB helicase from *Helicobacter pylori* (6 × 59 kDa) with 488 residues per monomer poses a big challenge at 850 MHz, already for assignment. Divide-and-conquer approaches (Wiegand et al. 2016a, b) or segmental isotope labeling of individual protein domains have thus been applied (Wiegand 2018), however without reaching close-to-complete assignment (around 70% of the N–terminal and 60% of the C-terminal domain were assigned.) Fig. 3 shows the NMR spectra collected on DnaB complexed with ADP:AlF_4_^−^ and single-stranded DNA (Wiegand et al. 2019). Figure 3a displays the ^13^C-detected 1D CP-spectra recorded at three different magnetic field strengths: 500 MHz, 850 MHz and 1200 MHz. The efficiency of the CP at 1200 MHz suffers again from offset effects, even more than in Fig. 1a, as the higher salt content of the sample (130 mM NaCl) reduces the rf-field strengths that can be safely applied according to the manufacturer. Figure 3b shows the 20 ms DARR spectra recorded at these different magnetic-field strengths. The increase in resolution with magnetic field is obvious (Fig. 3b, c). We have automatically picked the resonances in the aliphatic region (using CCPNmr (Fogh et al. 2002; Vranken et al. 2005)) of the 2D spectra and find an increase from 203 to 322 peaks between the 850 and 1200 MHz spectra (see Figure S4), highlighting the gain in resolution. This gain will allow us to go further in structural studies of this protein using 3D and 4D spectra to further increase resolution. While a crude measure of resolution, the number of picked peaks gives a simple quantitative proxy.

**Fig. 4:**
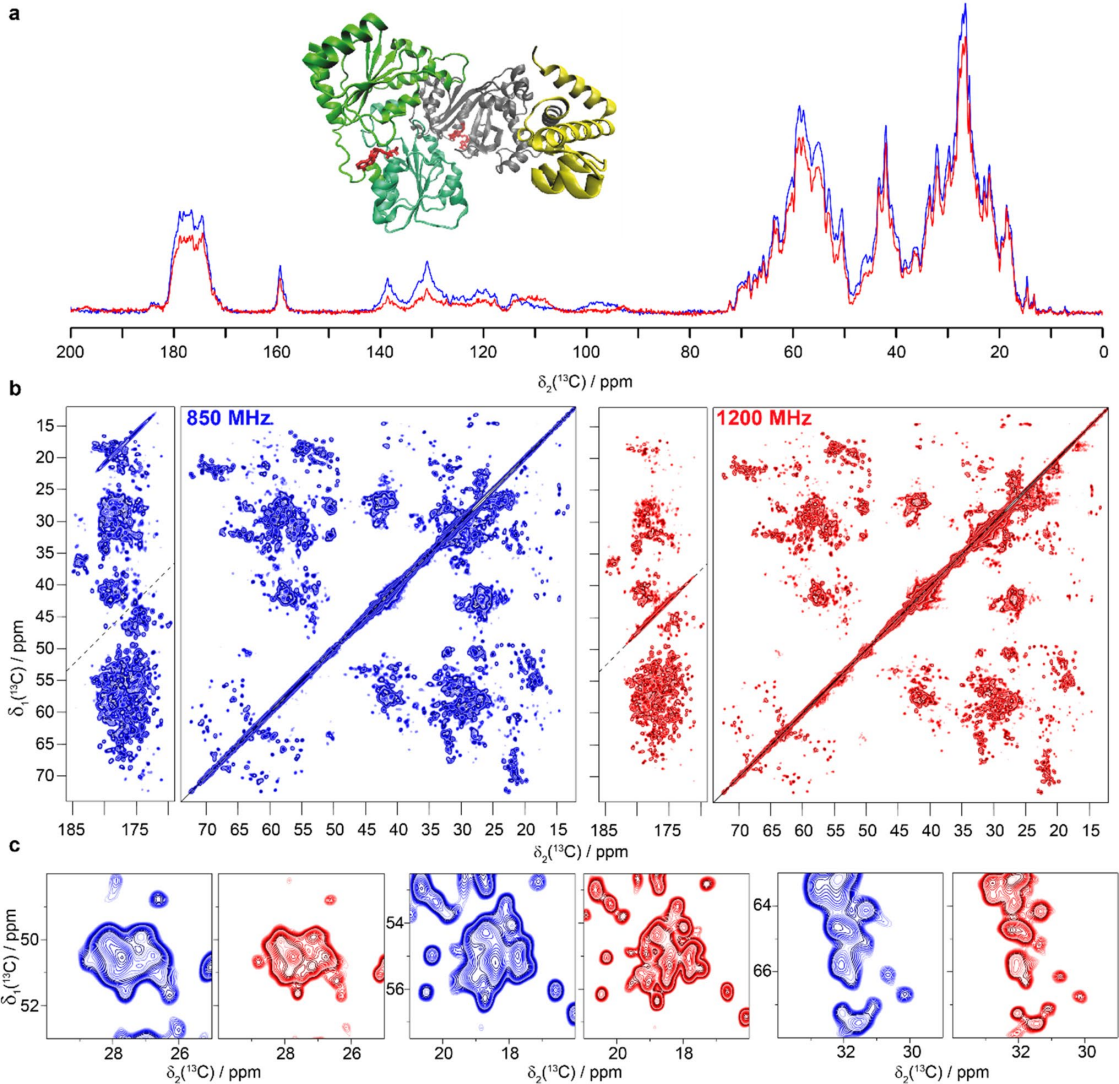
The RNA helicase and acetyltransferase TmcA. **a** Structural model (PDB ID: 2ZPA) (Chimnaronk et al. 2009) and 1D ^13^C-detected CP-MAS spectrum, **b** 20 ms DARR spectra and **c** expanded regions from the spectra in **b**. Spectra colored in blue were recorded at 850 MHz and spectra in red were measured at 1200 MHz. CP was matched at 70 and 44 kHz for ^1^H and ^13^C at 1200 MHz and at 60 and 43 kHz at 850 MHz

As an example for an even larger protein with 671 amino-acid residues, we studied the RNA helicase and acetyltransferase TmcA (Fig. 4) (Ikeuchi et al. 2008; Chimnaronk et al. 2009). 3.2 mm E-free probe and experiments at 1200 MHz might open up the possibility of at least partial assignments as suggested by the demonstrated gain in resolution.

**Fig. 5:**
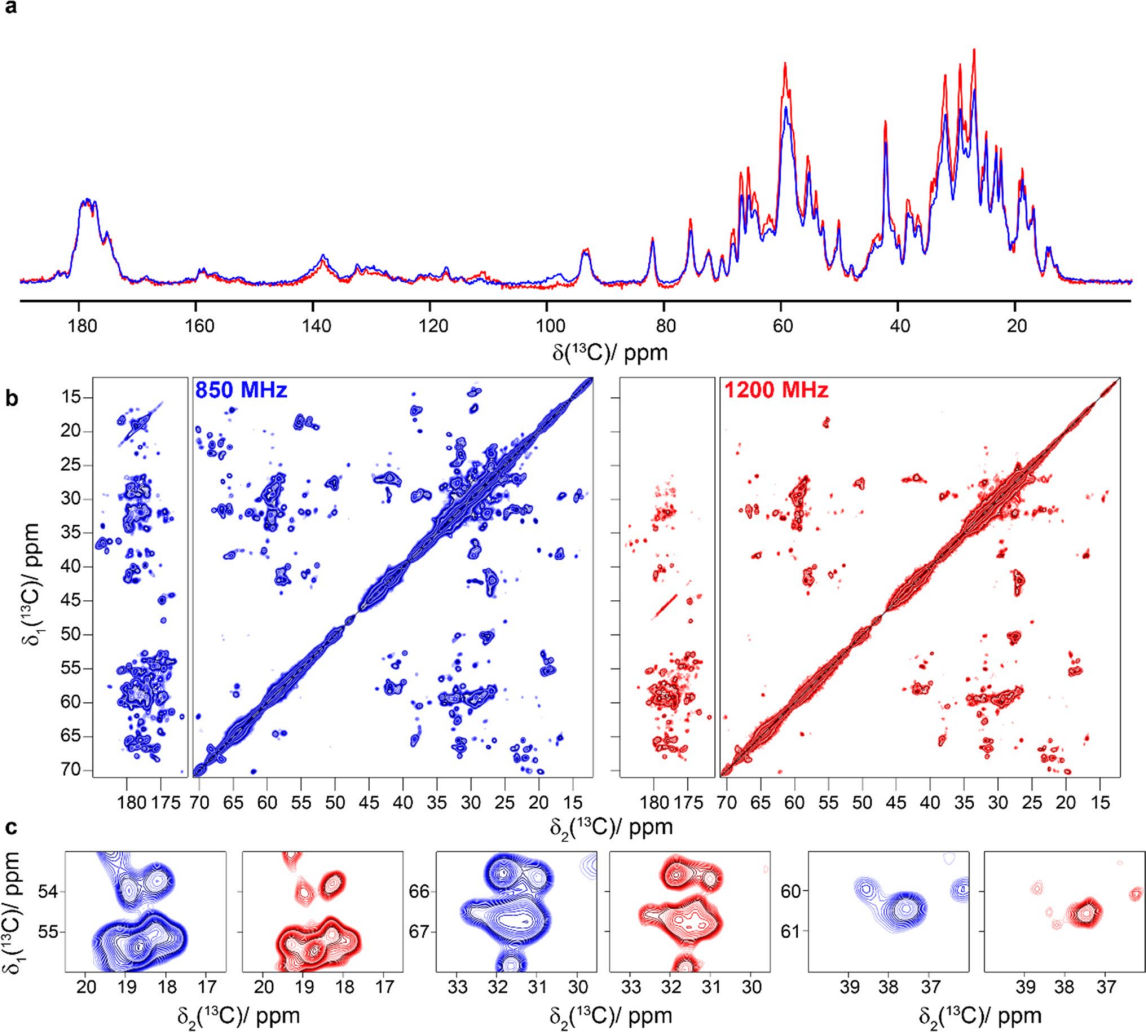
The African cichlid nackednavirus capsid ACNDVc. **a** 1D ^13^C-detected CP-MAS spectrum, **b** 20 ms DARR spectra and **c** expanded regions from the spectra in **b**. Spectra colored in blue were recorded at 850 MHz and spectra in red were measured at 1200 MHz. CP was matched at 75 and 47 kHz for ^1^H and ^13^C at 1200 MHz and at 60 and 41 kHz at 850 MHz

Finally, we measured the CP and DARR spectra of the viral capsid of the African chichlid nackednavirus, a non-enveloped fish virus and member of the Hepatitis B family (Lauber et al. 2017). The core protein constituting the capsid consists of 175 amino-acid residues. The corresponding spectra are shown in Fig. 5. The T = 3 icosahedral capsid is formed by 60 copies of A, B and C subunits that constitute the asymmetric unit. Unlike in the case of the HBV capsid (Lecoq et al. 2018), the signals from the different subunits are not resolved in the nackednavirus capsid spectra. They may, however, contribute to the heterogeneous line broadening that limits the resolution improvement when going to the higher field. As a consequence, the gain in spectral resolution is less spectacular than for the other samples.

### ^1^H-detected ^1^H–^15^N correlation spectroscopy

In addition to faster spinning (Penzel et al. 2019; Schledorn et al. 2020), higher fields can equally improve proton resolution (Xue et al. 2020). In samples where the linewidth is dominated by coherent homogeneous interactions, one can expect a linear improvement in resolution. Importantly, beyond this, further narrowing can be induced by the truncation of strong-coupling effects by the increased chemical-shift difference between the coupled protons at higher magnetic fields.

Strong coupling effects are important if the chemical-shift difference between two spins *m* and *n* is smaller than the second-order residual dipolar terms that include *m* and *n* (Malär et al. 2019b). In this case, the two resonances are not clearly separated within the dipolar linewidth. This effect is often most relevant for CH_2_ groups which have a strong dipolar coupling, and the chemical shifts of neighboring CH_2_ protons, in particular in the side-chains, are much closer than for example H_N_ or H$\alpha$ protons.

**Fig. 6:**
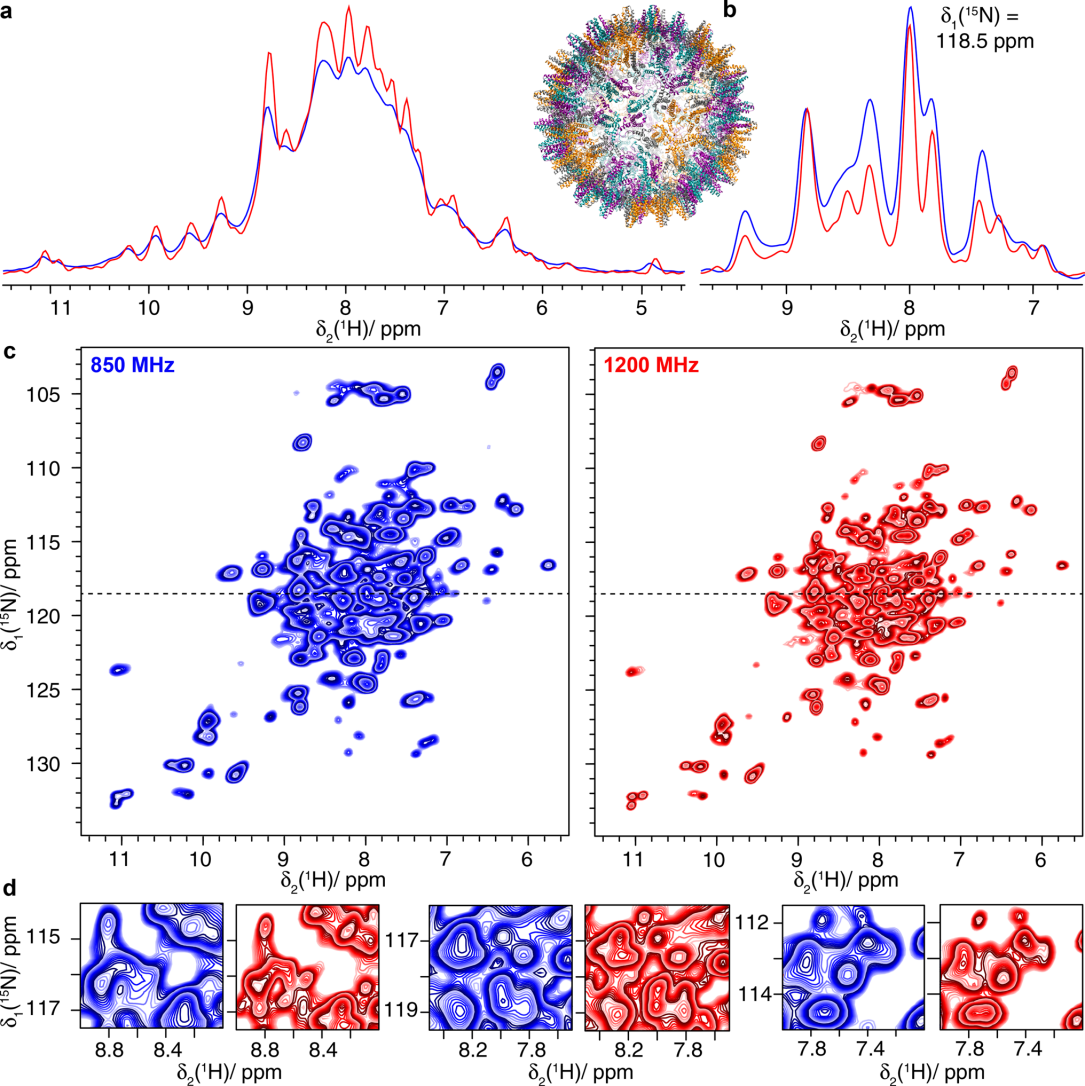
The Hepatitis B Virus Capsid dCp149. **a** 1D-hnH spectra and structural model (PDB ID:1QGT) (Wynne et al. 1999), **b** one-dimensional trace at δ_1_(^15^N) = 118.5 ppm of **c** 2D hNH spectra and **d** expanded regions from the spectra in **c**. Spectra colored in blue were recorded at 850 MHz and spectra in red were measured at 1200 MHz

We first investigated the Hepatitis B virus (HBV) nucleocapsid, composed of 240 copies of the core protein (Cp) (Wynne et al. 1999; Lecoq et al. 2019). Cp149 is a truncated version containing only the assembly domain. The 2D hNH spectra of ^2^H–^13^C–^15^N labeled and re-protonated Cp149 (dCp149) capsids are shown in Fig. 6 and demonstrate the increase in resolution at the higher field. This can be visualized by comparing the 2D hNH spectra and expanded regions shown in Fig. 6c and d, respectively, as well as in the one-dimensional trace at δ_1_(^15^N) = 118.5 ppm (Fig. 6b). Automated peak picking (using CCPNmr) performed in the amide region of the 2D spectra shows an increase from 110 to 157 peaks when going from 850 to 1200 MHz (see Figure S5). As for the carbon-detected spectra, the number of picked peaks in the ^1^H-detected spectra should only be used as a simple quantitative proxy for the resolution. As discussed for the ^13^C spectra of the nackednavirus, the resolution may be limited by unresolved peak splittings due to the presence of four molecules per asymmetric unit (Lecoq et al. 2019).

Investigations of integral membrane proteins in lipids still remain at the very edge of what is possible at current magnetic fields, because of their limited chemical-shift dispersion due to their mainly $\alpha$-helical secondary structure (David et al. 2018). We recently studied the cell-free synthesized hepatitis C virus (HCV) nonstructural membrane protein 4B (NS4B) (Jirasko et al. 2020). NS4B has a sequence length of 261 amino-acid residues, and is an oligomeric $\alpha$-helical integral membrane protein constituted of three subdomains (Gouttenoire et al. 2014). Even though multidimensional spectra could be obtained, sequential assignments could only be achieved for few residues (Jirasko et al. 2020).

**Fig. 7:**
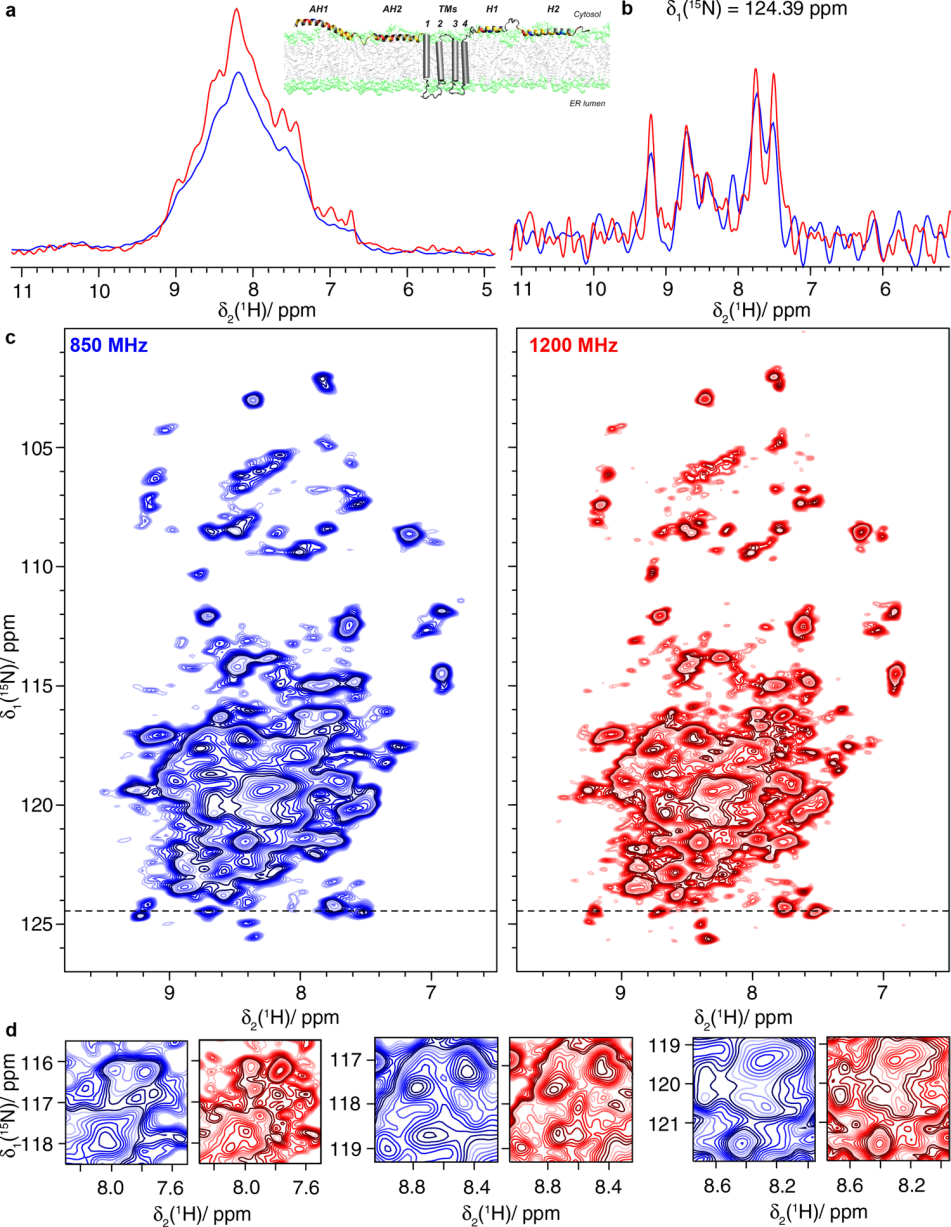
The hepatitis C virus non-structural protein dNS4B. **a** 1D-hnH spectra and structural model based on (Gouttenoire et al. 2014), **b** one-dimensional trace at δ_1_(^15^N) = 124.39 ppm of **c** 2D hNH spectra and **d** expanded regions from the spectra in **c**. Spectra colored in blue were recorded at 850 MHz and spectra in red were measured at 1200 MHz. MAS at 100 kHz was used

Figure 7 shows the 2D hNH spectra of ^2^H–^13^C–^15^N labeled NS4B (dNS4B). A clear gain in resolution is observed at 1200 MHz. This gain will enable further steps in the backbone assignment using 3D approaches, and might allow secondary-structure analysis sufficient for a critical evaluation of existing models.

**Fig. 8:**
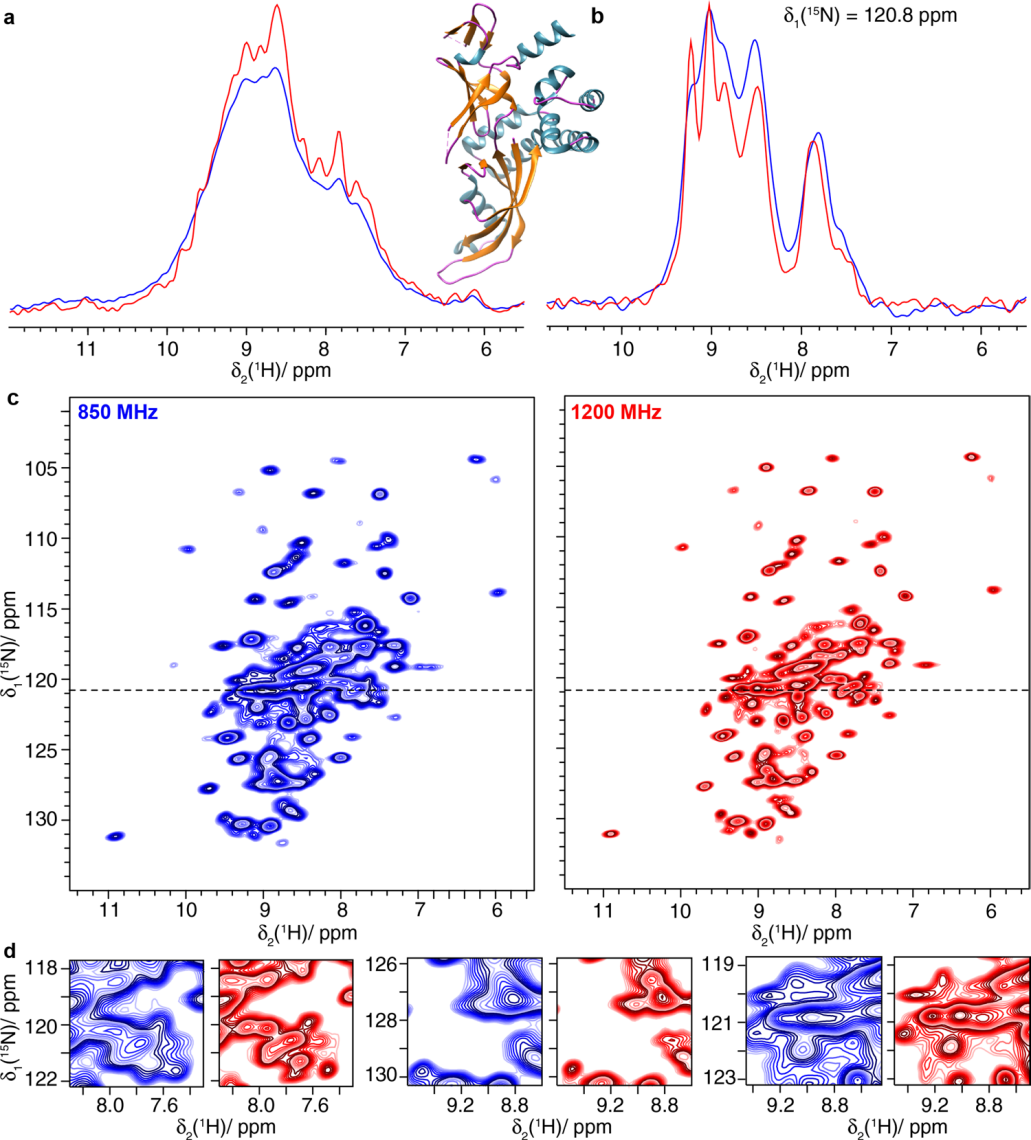
The Rpo4/7 protein complex (Rpo4C36S/Rpo7K123C). **a** 1D-hnH spectra and structural model of Rpo4/7 (PDB ID: 1GO3) (Todone et al. 2001), **b** one-dimensional trace at δ_1_(^15^N) = 120.8 ppm of **c** 2D hNH spectra and **d** expanded regions from the spectra in **c**. Spectra colored in blue were recorded at 850 MHz and spectra in red were measured at 1200 MHz. All experiments used 100 kHz MAS

We also recorded 2D hNH spectra on the ^13^C–^15^N labeled, and fully protonated, Rpo4/7 protein complex which is a subcomplex of archaeal RNA polymerase II (Fig. 8). Despite the full protonation, the spectra are highly resolved, and show higher resolution at 1200 MHz. Automated peak picking performed in the amide region of the 2D spectra shows an increase in the number of picked peaks (using CCPNmr) from 80 to 98 peaks when comparing 850 MHz to 1200 MHz (Figure S6).

**Fig. 9:**
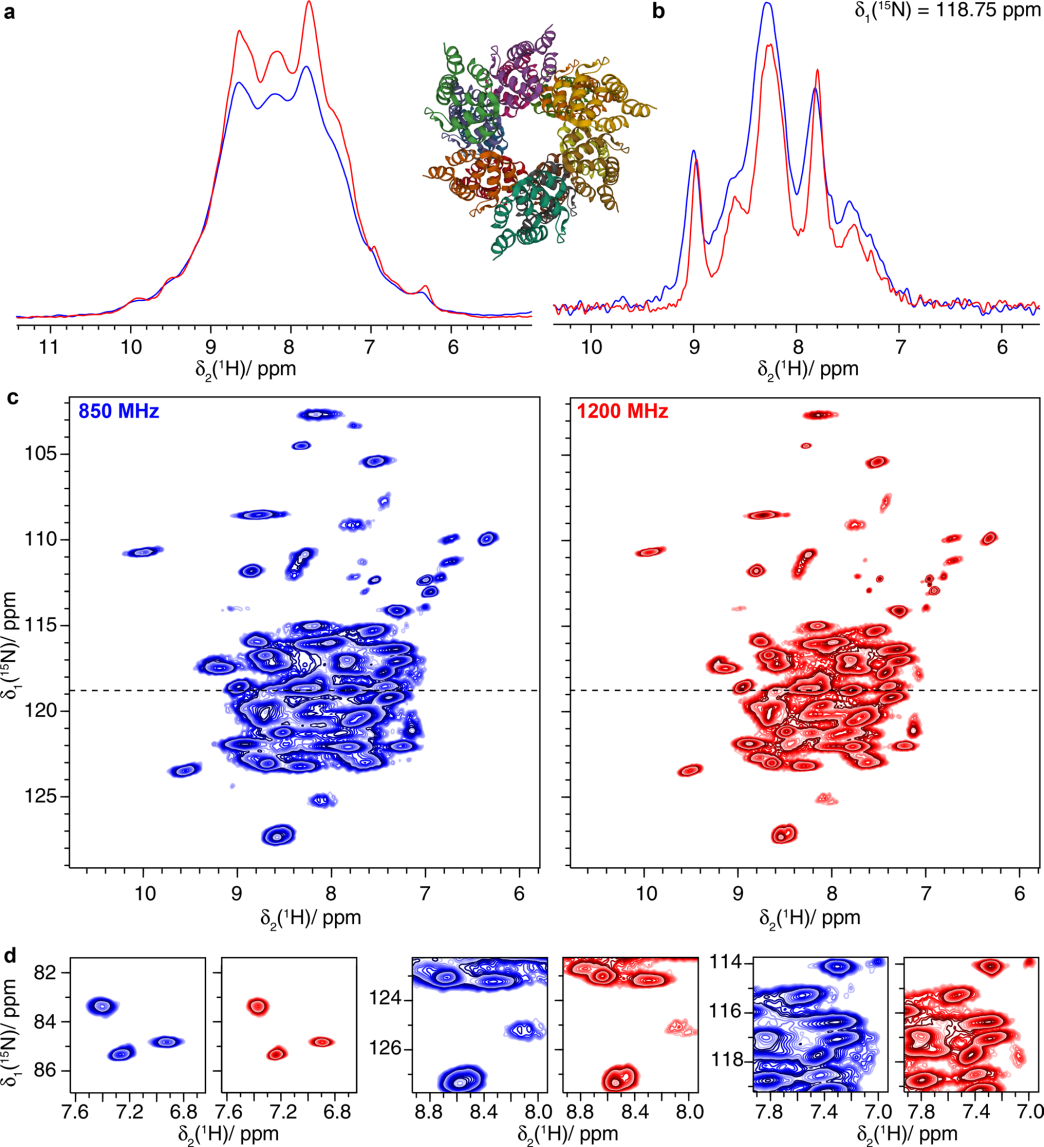
The filaments of PYRIN domain of mouse ASC. **a** 1D-hnH spectra and structural model of ASC filaments (PDB ID: 2N1F) (Sborgi et al. 2015), **b** one-dimensional trace at δ_1_(^15^N) = 118.75 ppm of **c** 2D hNH spectra and **d** expanded regions from the spectra in **c**. Spectra colored in blue were recorded at 850 MHz and spectra in red were measured at 1200 MHz. All experiments used 100 kHz MAS

Furthermore, ^1^H-detected 2D spectra were acquired on fully protonated and ^13^C–^15^N labeled ASC filaments (Fig. 9). The six $\alpha$-helices forming the monomer (Liepinsh et al. 2003; Sborgi et al. 2015; Ravotti et al. 2016) make it more challenging for solid-state NMR due to the narrower distribution of chemical shifts (see e.g. (David et al. 2018)) and broader lines (due to stronger dipolar couplings in $\alpha$-helices) (Malär et al. 2019b) compared to proteins with higher variety in secondary structure. The higher magnetic field brings improved resolution in 2D hNH spectra (Fig. 9a) also indicated by more peaks picked automatically (142 and 170 peaks in hNH spectrum at 850 MHz and 1200 MHz, respectively, Figure S7).

### ^1^H-detected ^1^H–^13^C correlation spectroscopy

**Fig. 10:**
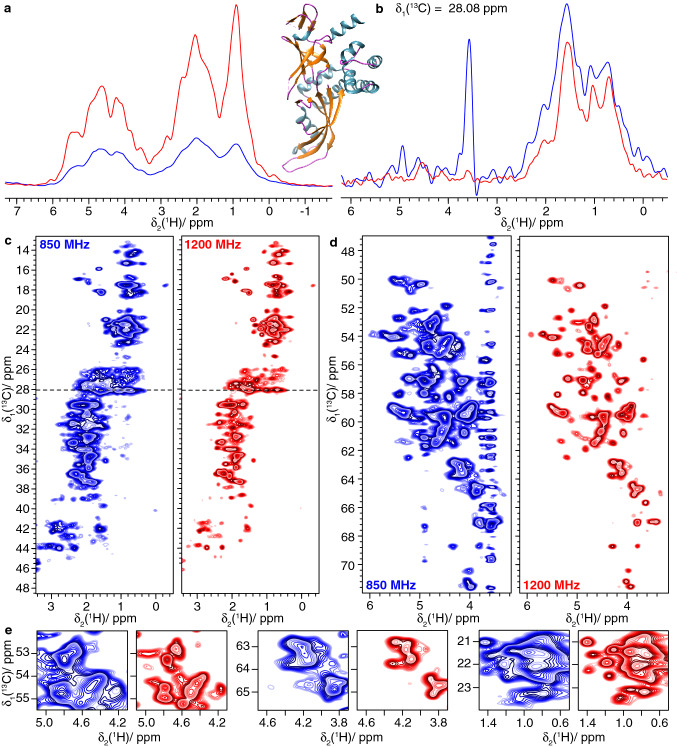
The Rpo4/7 protein complex (Rpo4C36S/Rpo7K123C). **a** 1D-hcH spectra and structural model of Rpo4/7 (PDB 1GO3) (Todone et al. 2001), **b** one-dimensional trace at δ_1_(^13^C)=28.08 ppm of **c**, **d** 2D hCH spectra and **e** expanded regions from the spectra in c-d. Spectra colored in blue were recorded at 850 MHz and spectra in red were measured at 1200 MHz

**Fig. 11:**
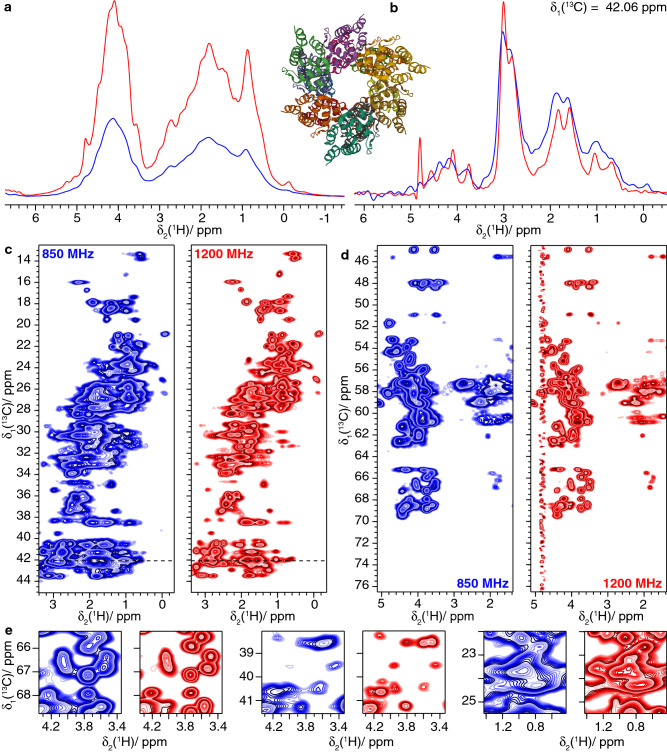
Filaments of the PYRIN domain of fully protonated mouse ASC. **a** 1D-hcH spectrum and structural model of ASC filaments (PDB 2N1F) (Sborgi et al. 2015), **b** one-dimensional trace at δ_1_(^13^C) = 42.06 ppm of **c**, **c**, **d** 2D hCH spectra and **e** expanded regions from the spectra in **c**, **d**. Spectra colored in blue were recorded at 850 MHz and spectra in red were measured at 1200 MHz

**Fig. 12:**
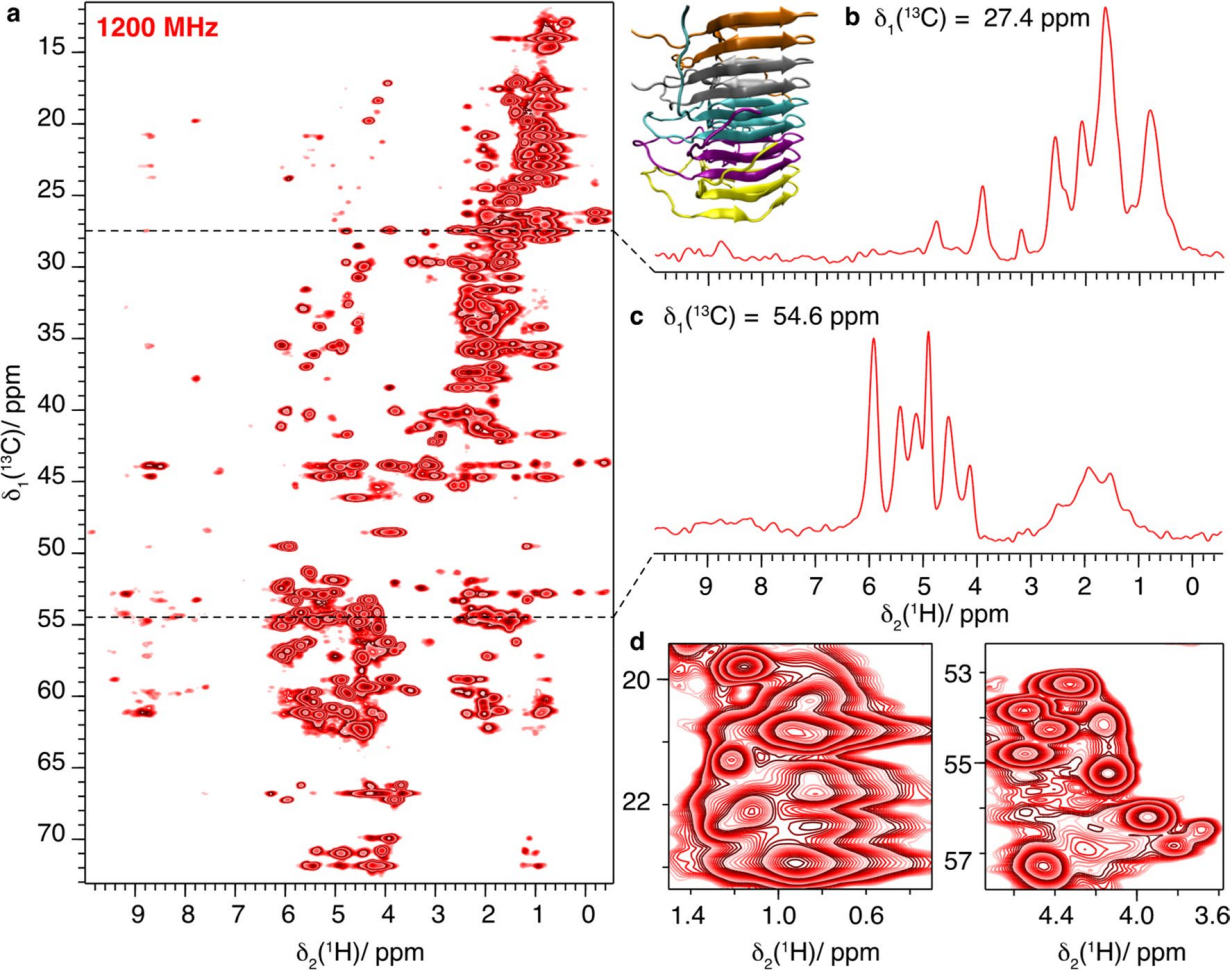
HET-s(218–289) fibrils fully protonated at 1200 MHz and 100 kHz MAS **a** 2D-hCH spectrum and structural model of HET-s(218–289)(PDB ID: 2RNM) (Wasmer et al. 2008), **b** one-dimensional trace at δ_1_(^13^C) = 118.75 ppm, **c** at 54.6 ppm and **d** expanded regions from the spectrum in **a**

A major limitation of proton-detected solid-state NMR spectroscopy today is the difficulty to observe H$\alpha$ and side-chain protons at high resolution. First, perdeuteration followed by back-exchange of the amide protons (and exchangeable side-chain protons) is often used to improve resolution, so that solely H_N_ are present in the sample. Selective labeling allows to introduce protons at selected side-chain positions (Kainosho et al. 2006; Asami and Reif 2012; Lacabanne et al. 2017; Movellan et al. 2019; Fogeron et al. 2021), but can make protein expression more complex and expensive. Detection of H$\alpha$ and side-chain protons is of course possible in fully protonated samples, but with an increased line width, resulting in poorer sensitivity and resolution. Narrower lines, at the expense of sensitivity, can be obtained by isotopic dilution (Asami and Reif 2012; Agarwal and Reif 2008; Agarwal et al. 2014). While H$\alpha$ and CH_3_ could often be resolved already at lower fields in particular in smaller proteins, CH_2_ remained in many cases severely broadened, as well as poorly dispersed due to the often highly similar chemical shifts of CH_2_ protons (Struppe et al. 2017). To illustrate the benefits of higher magnetic fields on fully protonated samples in this context, we recorded hCH spectra on three protein systems: ASC, Rpo4/7 and HET-s(218–289). The spectra are shown in Figs. 10, 11 and 12. For ASC and Rpo4/7, the 1200 MHz spectra are compared to spectra recorded at 850 MHz in Figs. 10 and 11 panels c–e. The traces shown in Figs. 10b and 11b illustrate the gain in resolution in the aliphatic sidechain region.

At higher field, a substantial gain in resolution is observed (Figs. 10a and 11a) in the hCH spectra. The improvements are clearly visible for the 2D hCH spectra in Figs. 10c and d and 11c and d (compare respective expanded regions and trace along F2 in Figs. 10b and 11b), and especially for the CH_2_ resonance region (in the range between 1 to 4 ppm for protons and 25 and 50 ppm for carbon) shown in Figs. 10c and 11c.

For HET-s, only a 1200 MHz spectrum was recorded, and is shown in Fig. 12. The excellent SNR obtained on this small protein allows even to detect further correlations than only one-bond. Similar observations are e.g. observed in (Friedrich et al. 2020). The spin diffusion under spin-lock condition during the CP actually seems to provide efficient polarization transfer, despite the scaling of the homonuclear dipolar couplings by a factor of −0.5 (Rhim et al. 1970). This is due to the absence of chemical-shift differences in the rotating frame leading to a strong-coupling situation for the homonuclear dipolar couplings, corresponding to the laboratory-frame situation for almost degenerate chemical shifts. (Xue et al. 2018)

In order to quantify the improvement in resolution when going to higher field, we compared the proton linewidths of ten randomly selected isolated peaks at 850 and 1200 MHz (Δ_ppm_(1200)/ Δ_ppm_(850), Figure S8). We observe that the improvement on the ppm scale is of about the expected value of 0.71 obtained from the field ratio. In the 2D hCH spectra recorded on the fully protonated samples a larger improvement is achieved, probably due to the more pronounced narrowing effect from the increased chemical-shift separation at higher field. The measurement of the bulk amide proton *T*_2_’(^1^H_N_) relaxation times (in Table S5) supports systematically the presence of this effect for all samples.

Access to information on side-chains is very important in sequential assignments, since notably the ^13^C shifts allow to identify amino-acid types. These frequencies were however difficult to observe when ^1^H-detection is used with deuterated proteins, since polarization transfer had to start on the H_N_, to go all the way out to side-chains carbons, and back to be detected. In protonated systems, CH_2_ were often too broad to be of use; this clearly is improved at highest available fields and fastest spinning frequencies. 3D versions of these spectra e.g. hCCH spectra, shall thus facilitate assignments of the aliphatic resonances at high field.

Distances between side-chain atoms are also very welcome as structural restraints; the better resolution can add more CH and CH_2_ groups to the already used CH_3_ protons in selectively labeled samples (Agarwal and Reif 2008; Agarwal et al. 2014; Xue et al. 2018). Interestingly, the better resolution also paves the way for the detailed analysis of side-chain dynamics; typically, ^13^C relaxation measurements become possible above 60 kHz MAS (Smith et al. 2016) because ^13^C spin diffusion is sufficiently suppressed. However, ^13^C detection is arduous at these MAS frequencies, and proton detection delivers better sensitivity. The increased resolution in the ^1^H dimension, at higher field, is therefore an important advantage in this context.

## Conclusions

We herein presented our first protein solid-state NMR spectra recorded at 1200 MHz revealing a significant gain in resolution for a variety of protein samples, ranging from amyloid fibrils, viral capsid proteins, protein complexes to helicases using ^13^C-, as well as ^1^H-detected experiments. For the samples described here, the improvement in resolution is variable but present for all samples.

The gain in resolution for ^13^C-detected spectra of large proteins will push the current resonance assignment limitations due to reduced spectral overlap and thereby provides an alternative to laborious biochemical approaches, for example segmental isotope labelling, or time-consuming spectroscopic methods such as 4D and 5D spectra. It will further allow studying structural and dynamic changes of high-molecular weight proteins upon interaction with other proteins, nucleic acids or small-molecule drugs, since the fate of more isolated peaks can be followed.

For ^1^H-detected spectra, we observe an increase in proton resolution resulting from the increased chemical-shift dispersion as well as the reduced coherent contribution to the line width at higher magnetic field. This effect is stronger for protonated samples, due to their denser proton dipolar network. The high field thus allows to better resolve aliphatic side-chain resonances and to characterize their structural properties as well as the dynamics. Importantly, the improved resolution and the concomitant gain in sensitivity at 1200 MHz, notably for the CH_2_ and CH_3_ groups, creates several new spectroscopic opportunities. First, side-chain resonances central in amino-acid identification and sequential assignments become more conveniently accessible. Second, the measurement of distance restraints involving side-chain atoms comes into reach also for uniformly labeled samples of large proteins. Last but not least, it renders the investigation of side-chain dynamics via ^13^ C relaxation a realistic objective.

## Materials and methods

### Sample preparation

^13^C–^15^N labeled protein samples were prepared as described in the literature: HET-s(218–289) fibrils (van Melckebeke et al. 2010), DnaB complexed with ADP:AlF_4_^−^ and DNA (Wiegand et al. 2019), Rpo4/7 protein complex of two subunits of RNA polymerase II (Torosyan et al. 2019) and filaments of PYRIN domain of mouse apoptosis-associated speck-like (ASC) protein containing a caspase-recruitment domain (Sborgi et al. 2015; Ravotti et al. 2016). The detailed protocols for TmcA, type 1 pili and ACNDVc will be described in forthcoming publications. ^2^H–^13^C–^15^N labeled and 100% re-protonated Hepatitis B Virus Capsid (dCp149) was prepared as described by (Lecoq et al. 2019) while ^2^H–^13^C–^15^N labeled NS4B (dNS4B) was synthesized in H_2_O (Jirasko et al. 2020).

### ^13^C-dectected spectroscopy

Solid-state NMR spectra were acquired on a wide-bore 850 MHz Bruker Avance III and on a standard-bore 1200 MHz Bruker Avance NEO spectrometer. ^13^C-detected solid-state NMR spectra were recorded using 3.2 mm Bruker Biospin “E-free probes”. The MAS frequency was set to 17.0 and 20.0 kHz at 850 MHz and 1200 MHz, respectively. The sample temperature was set to 278 K using the water line as an internal reference (Böckmann et al. 2009). The 2D spectra were processed with the software TOPSPIN (version 3.5 and 4.0.6, Bruker Biospin) with a shifted (3.0) squared cosine apodization function and automated baseline correction in the indirect and direct dimension. For further experimental details see Table S1.

### ^1^H-detected spectroscopy

The ^1^H detected spectra were acquired at 100 kHz MAS frequency using a Bruker 0.7 mm triple-resonance probe. The magic angle has been adjusted “on sample” by measuring *T*_2_’ proton transverse relaxation times and adjustment of the magic angle until the longest relaxation times were obtained (see Figure S2). The sample temperature was set to 293 K as determined from the supernatant water resonance (Gottlieb et al. 1997; Böckmann et al. 2009). Two-dimensional (2D) fingerprint spectra (hNH) were recorded on dCp149, dNS4B Rpo4/7 protein complex and ASC, and 2D-hCH spectra on the Rpo4/7 protein complex, ASC filaments and HET-s(218–289). At both spectrometer frequencies, the 2D spectra were recorded with identical acquisition parameters for each protein sample (see Tables S2 to S4). All ^1^H detected spectra were processed using Topspin 4.0.6 (Bruker Topspin) with zero filling to the double amount of data points and a shifted sine-bell apodization function in direct and indirect dimensions with SSB = 2.5. The direct dimension was truncated to 12.9 ms during processing of measurements at both magnetic field strengths.

Spectral analysis was performed using CcpNmr Anlaysis 2.4.2 (Fogh et al. 2002; Vranken et al. 2005; Stevens et al. 2011). The spectra were referenced to 4,4-dimethyl-4-silapentane-1-sulfonic acid (DSS). One-dimensional spectra were scaled to the same noise level and 2D spectra to the same intensity level to compare spectra recorded at the two magnetic fields.

## Supplementary Information

Below is the link to the Supplementary Information.

Supplementary Information 1 (PDF 3589 kb)

## Data Availability

The datasets generated during and/or analysed during the current study are available from the corresponding author on reasonable request.
