# Supplementary material for: Biomolecular solid-state NMR spectroscopy at 1200 MHz: the gain in resolution
Source: J Biomol NMR. 2021 Jun 25;75(6-7):255–72. doi: 10.1007/s10858-021-00373-x (PMC8275511; doi:10.1007/s10858-021-00373-x)
Supplement: Supplementary file 1 — Supplementary Information 1 (PDF 3589 kb) [file 10858_2021_373_MOESM1_ESM.pdf]

Supplementary Information for

Biomolecular solid-state NMR spectroscopy at highest field: the gain in resolution at 1200 MHz

Morgane Callon[#,1], Alexander A. Malär[#,1], Sara Pfister[#,1], Václav Rímal [#,1], Marco E. Weber[#,1], Thomas Wiegand[#,1], Johannes Zehnder[#,1], Matías Chávez[1], Rajdeep Deb[1], Riccardo Cadalbert[1], Alexander Däpp[1], Marie-Laure Fogeron[2], Andreas Hunkeler[1], Lauriane Lecoq[2], Anahit Torosyan[1], Dawid Zyla[3], Rudolf Glockshuber[3], Stefanie Jonas[3], Michael Nassal[4], Matthias Ernst*[,1], Anja Böckmann*[,2], Beat H. Meier*[,1]

[1]*Physical Chemistry, ETH Zurich, 8093 Zurich, Switzerland*

[2]*Molecular Microbiology and Structural Biochemistry, UMR 5086 CNRS/Université de Lyon, 69367 Lyon, France*

[3]*Institute of Molecular Biology and Biophysics, ETH Zurich, 8093 Zurich, Switzerland*

[4]*Dept. of Medicine II / Molecular Biology, University of Freiburg*

Supplementary Figures

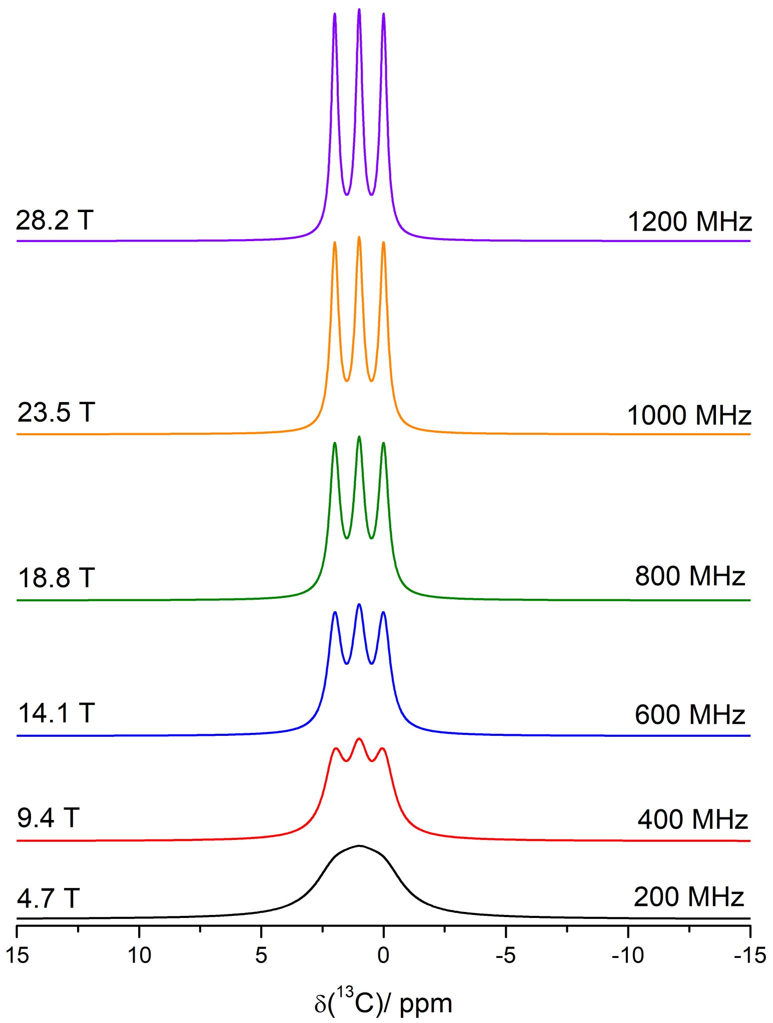

Figure S1: *NMR spectra gain in resolution on the ppm-scale by increasing the external magnetic field strength.* Simulated NMR spectra were obtained using SIMPSON.(Bak et al. 2000) The simulations were performed with three resonances centered at 0, 1 and 2 ppm, assuming a constant full-width at half maximum (FWHM= 100 Hz) and the magnetic field was varied in such simulations. The integral of the resonances on the ppm-scale is kept constant and Lorentzian lines are obtained.

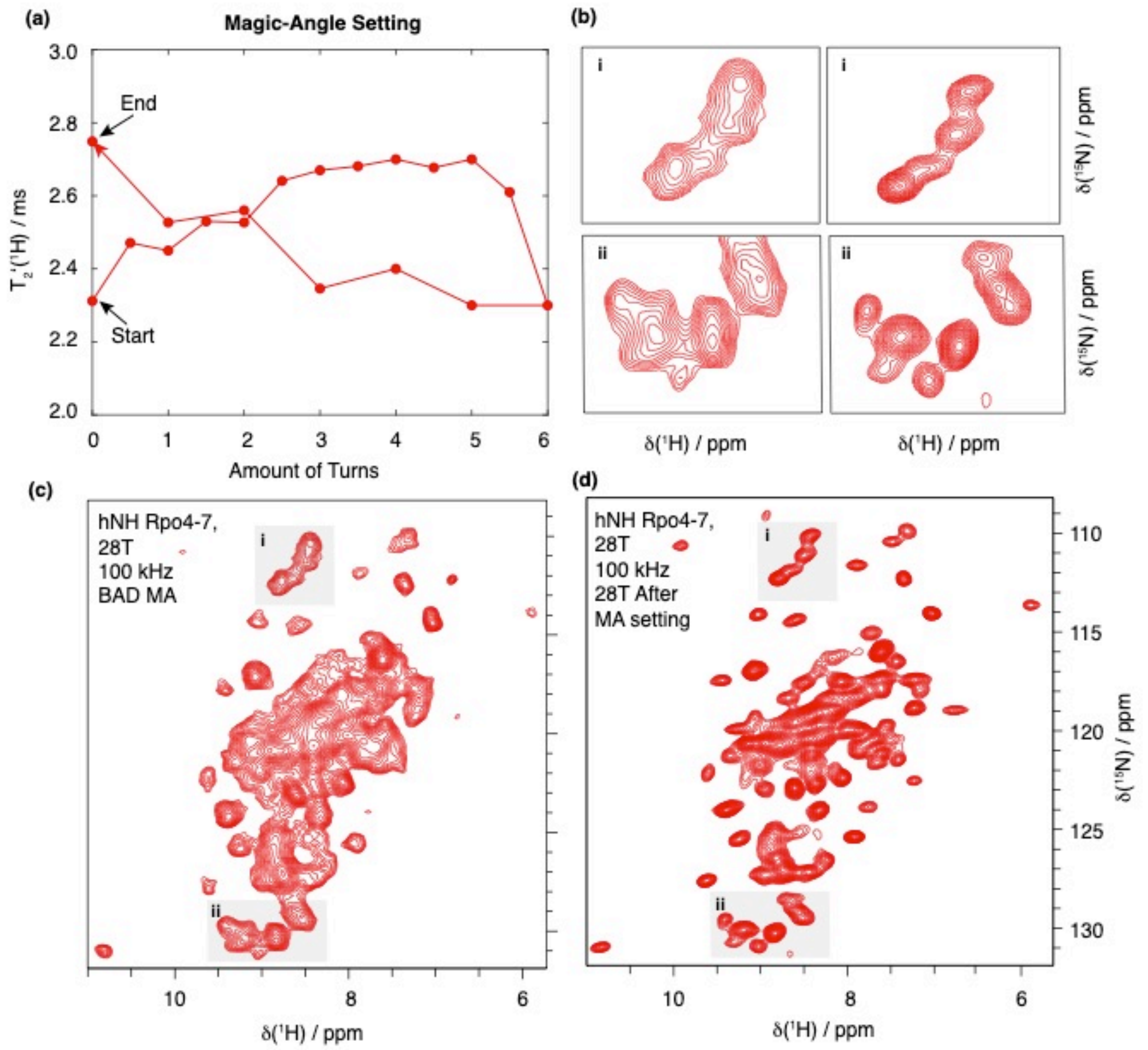

Figure S2: *"On sample" magic-angle setup by optimizing on the longest proton T_2' transverse relaxation times* **a** T_2'(^1H_N) transverse relaxation time as function of the number of turns of the magic-angle screw on a standard bore 0.7 mm probe-head. **b** zooms of the 2D hNH spectra of Rpo4/7 protein complex recorded **c** before and **d** after magic angle optimization with T_2'-on-sample method.

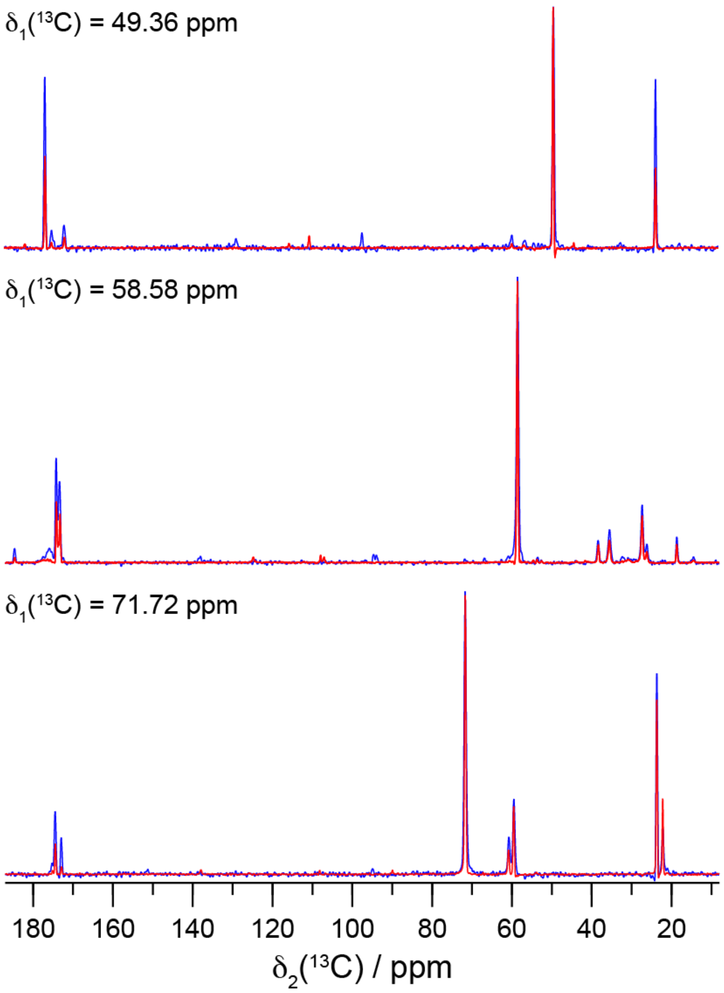

Figure S3: *The cross-peak intensities in 20 ms ^{13}C-^{13}C DARR is less at 1200 MHz compared to 850 MHz.* Three representative 1D traces along F2 taken from the DARR spectrum of HET-s(218-289) fibrils (Figure 2). The blue spectrum has been recorded at 850 MHz, the red spectrum at 1200 MHz. The spectra were scaled to the diagonal peak. In all cases, the cross-peak intensity at 1200 MHz is lower than at 850 MHz.

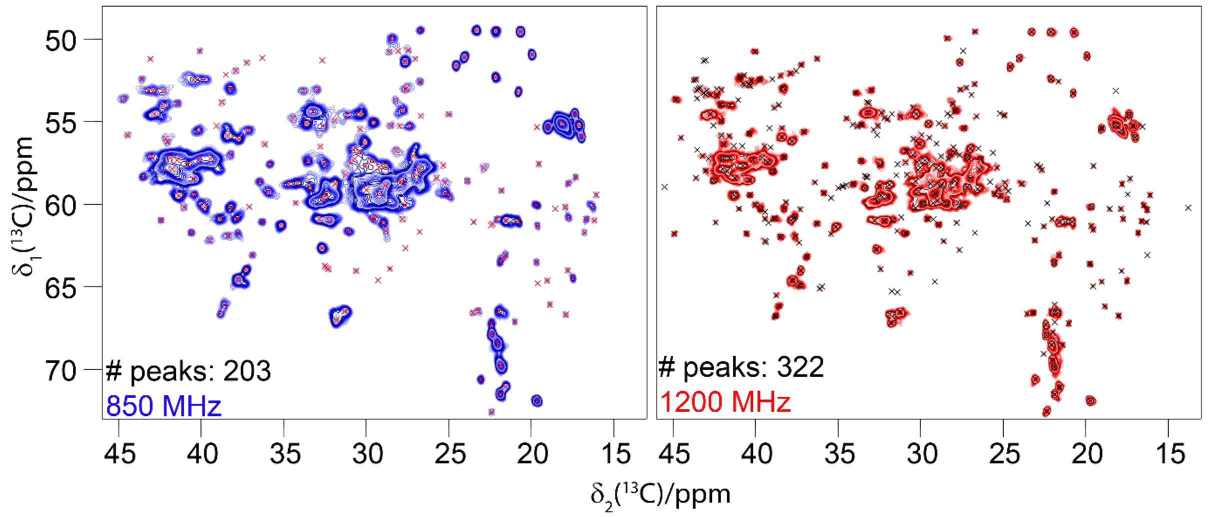

Figure S4: *The number of automatically picked peaks increases at higher magnetic-field strength.* Automatically picked resonances for DnaB (crosses) plotted on ^{13}C-^{13}C 20 ms DARR spectra recorded at 850 MHz (203 peaks) and 1200 MHz (322 peaks).

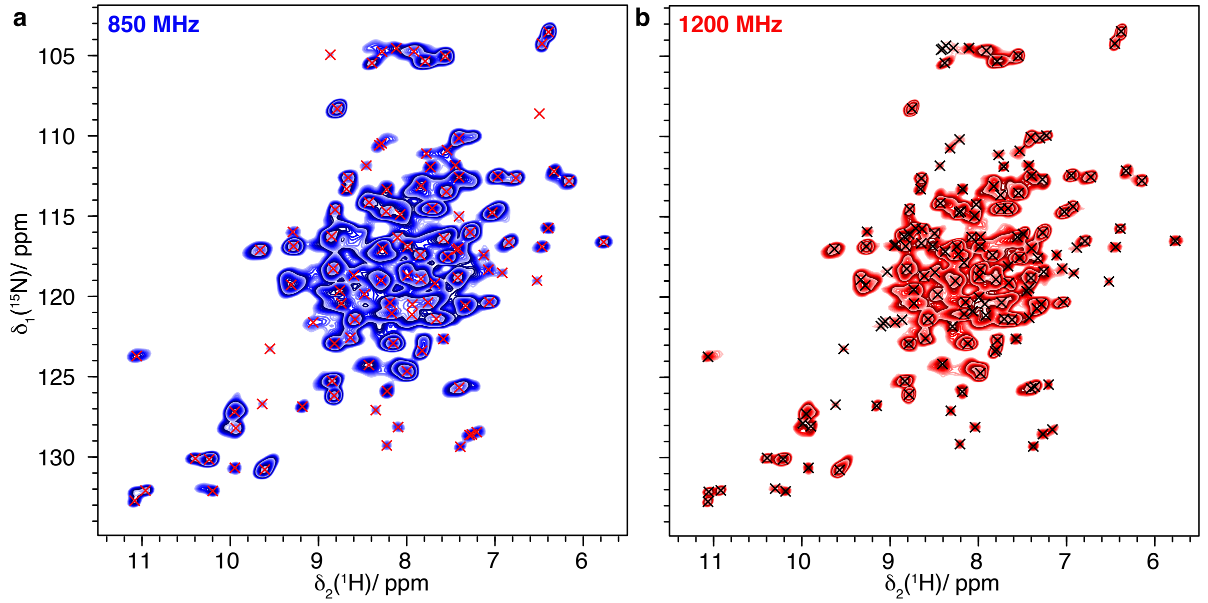

Figure S5: *The number of automatically picked peaks increases at higher magnetic-field strength.* Automatically picked resonances for dCp149 (crosses) plotted on 2D hNH spectra recorded at **a** 850 MHz (110 peaks) and **b** 1200 MHz (157 peaks). Note that number of peaks picked in the 1200 MHz spectrum is higher than the number of amino-acids in the protein due to the presence of four different molecules in the asymmetric unit of the T=4 icosahedral HBV capsid that causes peak splitting of Cp149 (Lecoq et al. 2018).

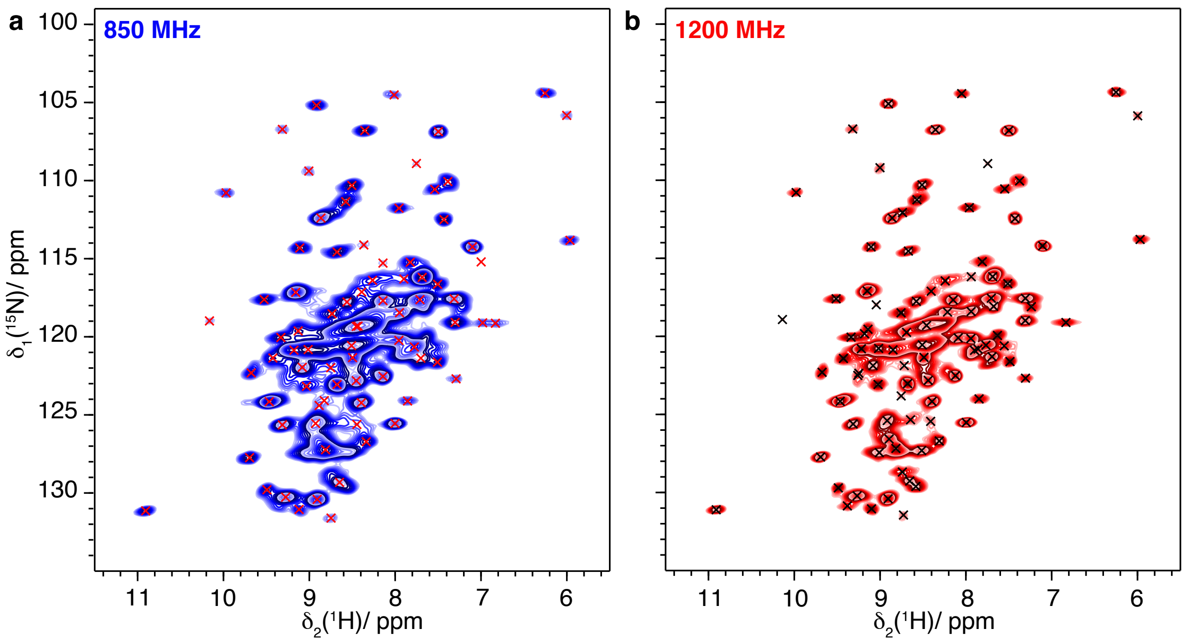

Figure S6: *The number of automatically picked peaks increases at higher magnetic-field strength.* Automatically picked resonances for the Rpo4/7 protein complex (Rpo4C36S/Rpo7K123C) (crosses) plotted on 2D hNH spectra recorded at **a** 850 MHz (80 peaks) and **b** 1200 MHz (98 peaks).

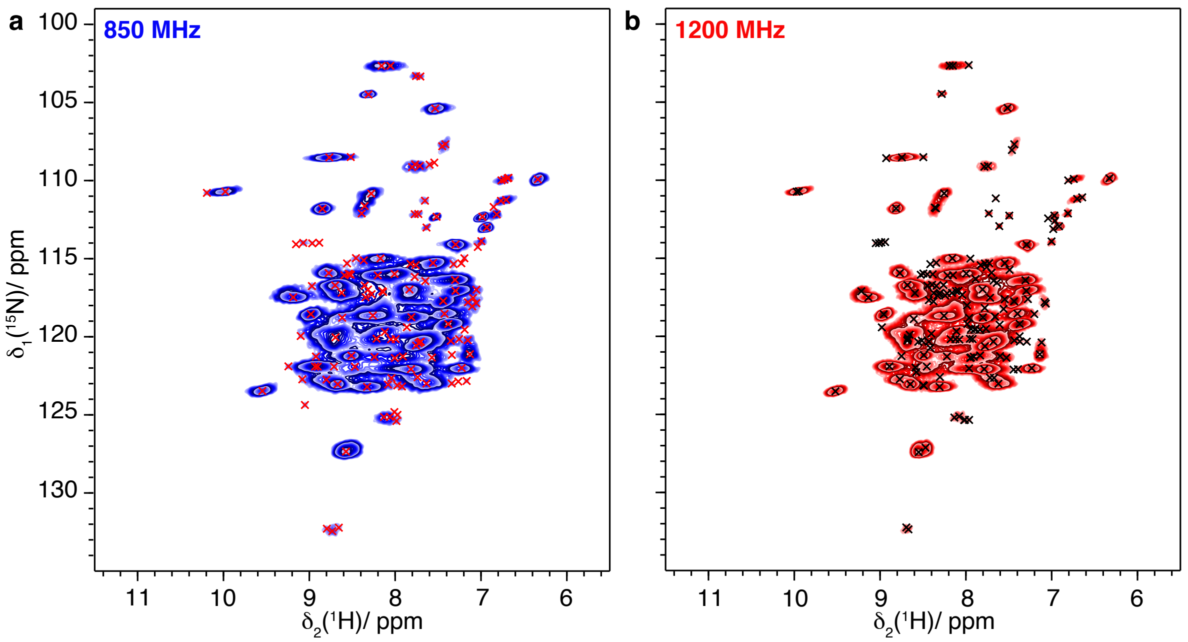

Figure S7: *The number of automatically picked peaks increases at higher magnetic-field strength.* Automatically picked resonances for the filaments of PYRIN domain of mouse ASC (crosses) plotted on 2D hNH spectra recorded at **a** 850 MHz (142 peaks) and **b** 1200 MHz (170 peaks).

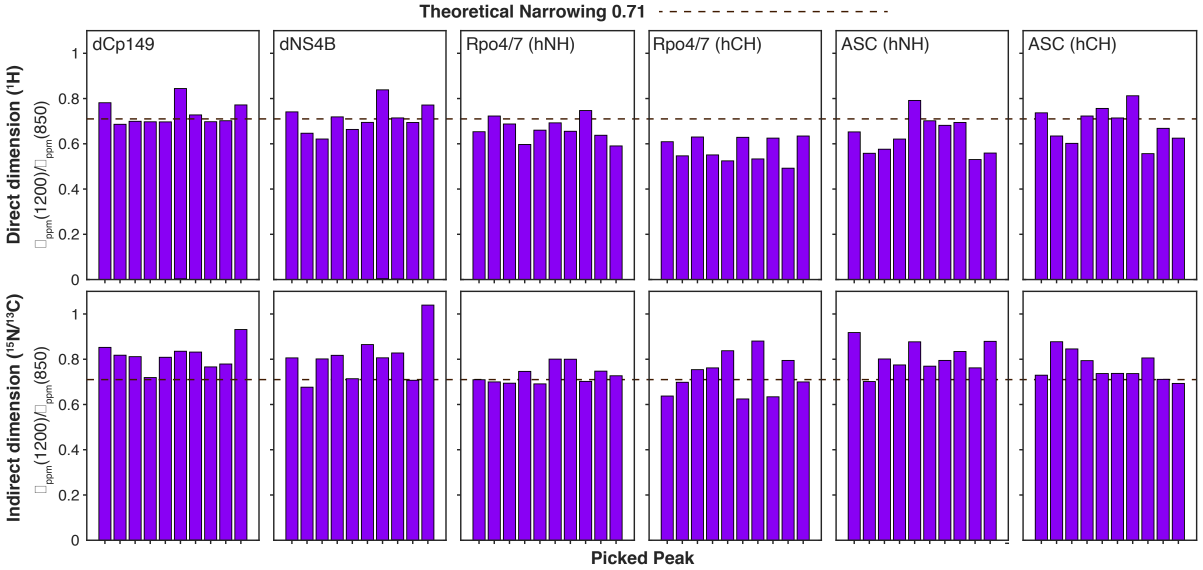

Figure S8: Comparison of the total linewidth ($\Delta_{ppm}(1200)$ / $\Delta_{ppm}(850)$) in ppm for both direct (^{1}H) and indirect dimensions (^{13}C or ^{15}N according to the experiment type) for a set of ten isolated and randomly selected peaks for the five different protein systems. The theoretical narrowing value (dashed black line) is obtained by calculating the field ratio 850/1200 = 0.71.

Supplementary Table 1: NMR parameters of 2D ^{13}C-^{13}C DARR correlation spectra recorded at 850 MHz (20.0 T) and 1200 MHz (28.2 T). ^{1}H hard pulses with a rf-field of 100 kHz (90 kHz) and for ^{13}C hard pulses with a rf-field of 50 kHz (42 kHz) were typically used at 850 MHz (1200 MHz).

**Experiment**	**DARR (DnaB)**			**DARR (HET-s(218-289))**	
MAS frequency/ kHz	17	17	20	17	20
Field/ T	11.7	20.0	28.2	20.0	28.2
Transfer I	HC-CP	HC-CP	HC-CP	HC-CP	HC-CP
^{1}H field/ kHz (power/ W)	60 (74)	60 (112)	55 (95)	60 (164)	75 (170)
X field/ kHz (power/ W)	43 (52)	43 (103)	29 (115)	43 (140)	48 (160)
Shape	Tangent ^{1}H	Tangent ^{1}H	Tangent ^{1}H	Tangent ^{1}H	Tangent ^{1}H
^{13}C carrier/ ppm	100	100	100	100	100
Time/ ms	0.5	0.5	0.6	0.6	0.6
Transfer II	DARR	DARR	DARR	DARR	DARR
^{1}H field/ kHz	17	17	20	17	20
Carrier/ ppm	100	100	100	100	100
Time/ ms	20	20	20	20	20
t1 increments	2560	2560	2560	2560	2560
Sweep width (t1)/ kHz	100	100	100	100	100
Acquisition time (t1)/ ms	12.8	12.8	12.8	12.8	12.8
t2 increments	3072	3072	3072	3072	3072
Sweep width (t2)/ kHz	100	100	100	100	100
Acquisition time (t2)/ ms	15.4	15.4	15.4	15.4	15.4
^{1}H Spinal64 decoupling power/ kHz	90	90	90	90	90
Interscan delay/ s	2.7	2.7	2.7	2.7	2.7
Number of scans	12	12	12	4	4
Measurement time/ h	23	23	23	8	8

Continue of Table 1: NMR parameters of 2D ^{13}C-^{13}C DARR correlation spectra and 1D ^{15}N,^{1}H CPMAS spectra recorded at 850 MHz (20.0 T) and 1200 MHz (28.2 T). ^{1}H hard pulses with a rf-field of 100 kHz (90 kHz) and for ^{13}C hard pulses with a rf-field of 50 kHz (42 kHz) were typically used at 850 MHz (1200 MHz).

**Experiment**	**DARR (TmcA)**		**DARR (Pili)**		**DARR (Nackednavirus)**	
MAS frequency/ kHz	17	20	17	20	17	20
Field/ T	20.0	28.2	20.0	28.2	20.0	28.2
Transfer I	HC-CP	HC-CP	HC-CP	HC-CP	HC-CP	HC-CP
^{1}H field/ kHz (power/W)	60 (115)	70 (160)	60 (104)	70 (127)	60 (111)	75 (188)
X field/ kHz (power/W)	43 (115)	44 (200)	43 (80)	44 (155)	41 (105)	47 (200)
Shape	Tangent ^{1}H	Tangent ^{1}H	Tangent ^{1}H	Tangent ^{1}H	Tangent ^{1}H	Tangent ^{1}H
^{13}C carrier/ ppm	100	100	100	100.5	100	100
Time/ ms	0.5	0.5	0.7	1.0	0.6	0.6
Transfer II	DARR	DARR	DARR	DARR	DARR	DARR
^{1}H field/ kHz	17	20	17	20	17	20
Carrier/ ppm	100	100	100	100	100	100
Time/ ms	20	20	20	20	20	20
t1 increments	2560	2560	2560	2560	2560	2560
Sweep width (t1)/ kHz	100	100	100	100	100	100
Acquisition time (t1)/ ms	12.8	12.8	12.8	12.8	12.8	12.8
t2 increments	3072	3072	3072	3072	3072	3072
Sweep width (t2)/ kHz	100	100	100	100	100	100
Acquisition time (t2)/ ms	15.4	15.4	15.4	15.4	15.4	15.4
^{1}H Spinal64 decoupling power/ kHz	90	90	90	90	90	90
Interscan delay/ s	2.7	2.7	2.7	2.7	2.7	2.7
Number of scans	12	12	8	8	8	8
Measurement time/ h	23	23	16	16	16	16

**Experiment**	**^{15}N,^{1}H CPMAS (HET-s(218-289))**	
MAS frequency/ kHz	17	20
Field/ T	20.0	28.2
Transfer I	HN-CP	HN-CP
^{1}H field/ kHz (power/W)	60 (106)	75 (170)
X field/ kHz (power/W)	44 (343)	35 (190)
Shape	Tangent ^{1}H	Tangent ^{1}H
Time/ ms	1.2	1.4
t2 increments	1024	1024
Sweep width (t2)/ kHz	100	100
Acquisition time (t2)/ ms	5.1	5.1
^{1}H Spinal64 decoupling power/ kHz	90	90
Interscan delay/ s	1.5	1.5

Number of scans	2	2
Measurement time/ s	6	6

Supplementary Table 2: NMR parameters of 2D hNH and hCH spectra recorded on ^{13}C-^{15}N Rpo4/7 protein complex sample at 850 MHz (20.0 T) and 1200 MHz (28.2 T). ^{1}H hard pulses with a rf-field of 150 kHz, ^{13}C hard pulses with 100 kHz and ^{15}N pulses with 62.5 kHz were used.

**Protein**	**Rpo4/7 (hNH)**		**Rpo4/7 (hCH)**	
Field / T	20	28	20	28
MAS frequency / kHz	100	100	100	100
Number of scans	48	48	40	40
t1 increment	524	740	724	1024
Sweep width (t1) / ppm	120	120	80	80
Acquisition time (t1) / ms	25.3	25.3	21.2	21.2
t2 increment	4096	4096	4096	4096
Sweep width (t2) / ppm	46.7	46.2	46.7	46.2
Acquisition time (t2) / ms	51.6	36.8	25.8	36.8
^{1}H dec (swftPPM) / kHz	10	10	10	10
^{15}N dec (WALTZ64) / kHz	5	5	-	-
^{13}C dec (WALTZ64) / kHz	-	-	5	5
Water sup. (120 ms) / kHz	20	20	20	20
Interscan delay / s	1.27	1.65	1.27	1.65
Experiment time	10h10	18h	11h30	20h50
**Transfer 1**	HN (dipolar)	HN (dipolar)	HC (dipolar)	HC (dipolar)
^{1}H field / kHz (power/ W)	83.8 (13)	85 (15.5)	83.8 (13)	76.6 (12)
^{15}N or ^{13}C field / kHz (power/ W)	13.9 (1.4)	22 (2)	18.5 (1)	20 (1.3)
Shape	Tangent ^{1}H	Tangent ^{1}H	Tangent ^{1}H	Tangent ^{1}H
Time / ms	1.4	1.4	0.7	0.7
**Transfer 2**	NH (dipolar)	NH (dipolar)	CH (dipolar)	CH (dipolar)
^{1}H field / kHz (power/ W)	80.6 (12)	86.6 (16)	73.5 (10)	76.6 (12)
^{15}N or ^{13}C field / kHz (power/ W)	13.9 (1.4)	22 (2)	20 (1)	20 (1.3)
Shape	Tangent ^{1}H	Tangent ^{1}H	Tangent ^{1}H	Tangent ^{1}H
Time / ms	1.0	0.9	0.7	0.7
Carrier ^{15}N / ppm	117.5	177.5	-	-
Carrier ^{13}C / ppm	-	-	40	40
Carrier ^{1}H / ppm	4.8	4.8	4.8	4.8

Supplementary Table 3: NMR parameters of 2D hNH and hCH spectra recorded on ^{13}C-^{15}N ASC and HET-s(218-289) samples at 850 MHz (20.0 T) and 1200 MHz (28.2 T). ^{1}H hard pulses with a rf-field of 150 kHz, ^{13}C hard pulses with 100 kHz and ^{15}N pulses with 62.5 kHz were used.

**Protein**	**ASC (hNH)**		**ASC (hCH)**		**HET-s (hCH)**
Field / T	20	28	20	28	28
MAS frequency / kHz	100	100	100	100	100
Number of scans	64	64	32	32	40
t1 increment	512	724	1536	2170	1024
Sweep width (t1) / ppm	70	70	180	180	80
Acquisition time (t1) / ms	42.5	42.5	20	20	21.2
t2 increment	2048	3072	2048	3072	4096
Sweep width (t2) / ppm	20	19.8	46.7	46.3	46.2
Acquisition time (t2) / ms	60.2	64.5	25.8	27.6	36.8
^{1}H dec (swftPPM) / kHz	10	10	10	10	10
^{15}N dec (WALTZ64) / kHz	5	5	-	-	-
^{13}C dec (WALTZ64) / kHz	-	-	5	5	5
Water sup. (120 ms) / kHz	20	20	20	20	20
Interscan delay / s	1	1.18	1	1.18	1.14
Experiment time	11h	18h41	15h51	27h03	15h35
**Transfer 1**	HN (dipolar)	HN (dipolar)	HC (dipolar)	HC (dipolar)	HC (dipolar)
^{1}H field / kHz (power/ W)	67.4 (10.5)	89.4 (13.7)	65.8 (10)	132.7 (30.2)	76.5 (12.5)
^{15}N or ^{13}C field / kHz (power/ W)	20.0 (4.51)	14.7 (2)	30.4 (5.175)	32.9 (3.5)	20 (1.3)
Shape	Tangent ^{1}H	Tangent ^{1}H	Tangent ^{1}H	Tangent ^{1}H	Tangent ^{1}H
Time / ms	1.5	1.3	0.7	0.7	0.7
**Transfer 2**	NH (dipolar)	NH (dipolar)	CH (dipolar)	CH (dipolar)	CH (dipolar)
^{1}H field / kHz (power/ W)	65.8 (10)	88.0 (13.3)	58.8 (8)	130.9 (29.4)	75 (12)
^{15}N or ^{13}C field / kHz (power/ W)	20.0 (4.51)	14.7 (2)	30.4 (5.175)	32.9 (3.5)	20 (1.3)
Shape	Tangent ^{1}H	Tangent ^{1}H	Tangent ^{1}H	Tangent ^{1}H	Tangent ^{1}H
Time / ms	1.5	1.3	0.7	0.7	0.7
Carrier ^{15}N / ppm	107	106.4	-	-	-
Carrier ^{13}C / ppm	-	-	58.5	58.0	40
Carrier ^{1}H / ppm	4.8	4.8	4.4	4.4	4.8

Supplementary Table 4: NMR parameters of 2D hNH spectra recorded on the deuterated samples (dCp149 and dNS4B) at 850 MHz (20.0 T) and 1200 MHz (28.2 T). ^{1}H hard pulses with a rf-field of 150 kHz and ^{15}N pulses with 62.5 kHz were used.

**Protein**	**dCp149**		**dNS4B**	
Field / T	20	28	20	28
MAS frequency / kHz	100	100	100	100
Number of scans	48	48	80	80
t1 increment	362	512	284	400
Sweep width (t1) / ppm	80	80	40	40
Acquisition time (t1) / ms	26.2	26.2	41.2	41.1
t2 increment	4096	4096	4096	4096
Sweep width (t2) / ppm	46.7	46.2	46.7	46.2
Acquisition time (t2) / ms	51.6	36.8	51.6	36.8
^{1}H dec (swftPPM) / kHz	10	10	10	10
^{15}N dec (WALTZ64) / kHz	5	5	5	5
Water sup. (120 ms) / kHz	20	20	20	20
Interscan delay / s	1.5	2.2	1.2	1.43
Experiment time	8h	16h30	8h50	14h22
**Transfer 1**	HN (dipolar)	HN (dipolar)	HN (dipolar)	HN (dipolar)
^{1}H field / kHz (power/ W)	79 (11)	81 (14)	82.2 (12)	87.5 (15)
^{15}N field / kHz (power/ W)	14 (1.4)	14 (2)	14 (1.4)	14.6 (2)
Shape	Tangent ^{1}H	Tangent ^{1}H	Tangent ^{1}H	Tangent ^{1}H
Time / ms	1.6	1.5	1.0	1.0
**Transfer 2**	NH (dipolar)	NH (dipolar)	NH (dipolar)	NH (dipolar)
^{1}H field / kHz (power/ W)	75.7 (10)	78.1 (13)	78.7 (11)	87.5 (15)
^{15}N field / kHz (power/ W)	14 (1.4)	14 (2)	14 (1.4)	14.6 (2)
Shape	Tangent ^{1}H	Tangent ^{1}H	Tangent ^{1}H	Tangent ^{1}H
Time / ms	1.8	1.7	1.2	1.2
Carrier ^{15}N / ppm	107	107	116.5	116.5
Carrier ^{1}H / ppm	4.8	4.8	4.8	4.8

Supplementary Table 5: amide proton transverse relaxation times T_2' (^1H_N) measured in ^{1}H-detected experiments at 850 MHz (20.0 T) and 1200 MHz (28.2 T). Proteins whose name starts with the letter d are perdeuterated and fully back-exchanged.

**Protein**	**dCp149**		**dNS4B**		**Rpo4/7**		**ASC**	
Field / T	20.0	28.2	20.0	28.2	20.0	28.2	20.0	28.2
T_2' (^1H_N) / ms	10.40	11.36	4.69	4.73	2.53	2.69	2.6	2.77

Supplementary References

Bak M, Rasmussen JT, Nielsen NC (2000) SIMPSON: A general simulation program for solid-state NMR spectroscopy. Journal Of Magnetic Resonance 147:296–330. https://doi.org/10.1006/jmre.2000.2179

Lecoq L, Wang S, Wiegand T, et al (2018) Localizing conformational hinges by NMR: Where do hepatitis b virus core proteins adapt for capsid assembly? Chemphyschem 19:1336–1340. https://doi.org/10.1002/cphc.201800211
